# Energy crisis in Europe enhances the sustainability of green chemicals†

**DOI:** 10.1039/d3gc01053h

**Published:** 2023-06-23

**Authors:** Abhinandan Nabera, Ioan-Robert Istrate, Antonio José Martín, Javier Pérez-Ramírez, Gonzalo Guillén-Gosálbez

**Affiliations:** a Institute for Chemical and Bioengineering, Department of Chemistry and Applied Biosciences ETH Zürich Vladimir-Prelog-Weg 1 Zürich 8093 Switzerland jpr@chem.ethz.ch gonzalo.guillen.gosalbez@chem.ethz.ch

## Abstract

Ammonia and methanol are essential to modern societies, but their production has been heavily reliant on natural gas, which contributes to supply disruptions and significant CO_2_ emissions. While low-carbon or green production routes have been extensively researched, their adoption has been hindered by higher costs, making them unsustainable. However, a recent energy crisis in Europe has created a unique opportunity to shift towards greener production technologies. Here we show that, green ammonia, produced through wind-powered water electrolysis, had the potential to outperform its fossil counterpart for six months as of December 2021, while methanol produced through CO_2_ capture and wind-based water electrolysis became an economically appealing alternative. With a coordinated effort from academia, industry, and policymakers, Europe can lead the grand transition towards more sustainable practices in the chemical industry.

## Abstract 2

Europe's energy crisis has made green routes for ammonia and methanol synthesis economically competitive. This presents an opportunity for Europe to lead the grand transition towards a sustainable chemical industry.

## Graphical abstract

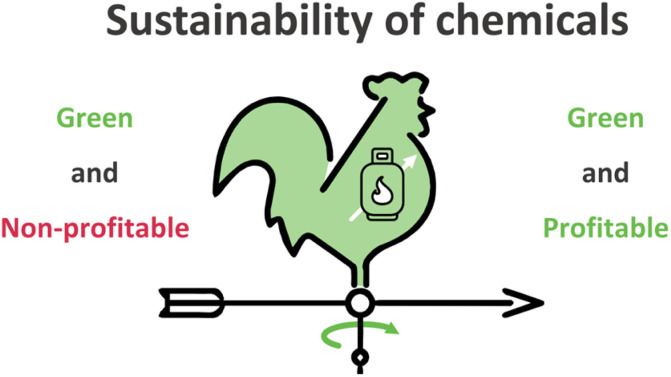

## Introduction

The chemical industry heavily relies on fossil fuels, making it the source of 5.6 Gt CO_2_-eq per year, comparable to approximately 10% of global anthropogenic greenhouse gas (GHG) emissions.^1,2^ Ammonia and methanol are key elements of the chemical industry, serving as building blocks for a myriad of valuable products. Ammonia plays a critical role in the manufacture of nitrogen fertilisers, which are essential for ensuring global food security.^3–5^ Consequently, the increasing population has led to a rise in the demand for ammonia. The global production volume of ammonia is approximately 185 Mt per year, resulting in roughly 0.5 Gt CO_2_-eq per year of emissions, equivalent to about 9% of the chemical sector's emissions.^6^ It is further estimated that by 2050, the demand for ammonia will increase to 355 Mt per year.^6^

On the other hand, methanol is primarily used as a fuel or fuel additive, offering cleaner combustion and lower emissions in comparison to fossil fuels.^7^ Moreover, the versatile nature of methanol allows it to act as an intermediate in the production of olefins and aromatics. It is also utilised to manufacture various compounds, including formic acid, dimethyl ether, and methylamine.^8–10^ With global methanol production currently at approximately 100 Mt per year, the corresponding life cycle GHG emissions are around 0.3 Gt CO_2_-eq per year, accounting for approximately 5% of the chemical sector's emissions.^11^ Projections indicate that the demand for methanol will rise to 120 Mt per year by 2025 and further escalate to 500 Mt per year by 2050.^12^

Between 60 and 70% of ammonia is produced from syngas obtained by steam reforming of natural gas.^6^ The Haber–Bosch process, which is energy-intensive and operates at demanding conditions (150–250 bar and 350–550 °C), is used to produce ammonia *via* reaction of nitrogen and hydrogen over iron-based catalysts.^13^ Green ammonia production is achievable through the green Haber–Bosch process, which involves using hydrogen generated from water electrolysis powered by renewable sources such as wind or solar.^6,14,15^ This hybrid electro- and thermo-catalytic scheme is currently in intense development and has been proposed as the first feasible low-emission alternative.^16^ The hydrogen required for the Haber–Bosch process can also be produced from renewable feedstock, *e.g.*, *via* biomass gasification. This route converts biomass, such as agricultural waste or wood chips, to syngas, which is further processed to produce hydrogen.^17^ A longer-term option, such as direct electrocatalytic reduction of nitrogen on a large or small scale, is highly desirable due to its association with renewable electricity sources. However, the early stage of development of this technology makes its industrial implementation currently impractical.^18^

Standard methanol production requires hydrogenation of CO over a Cu–Zn–Al catalyst at high pressure and mild temperature (35–100 bar and 200–300 °C).^19^ Similar to ammonia, 65% of global methanol relies on natural gas.^11^ With regard to the production of green methanol, the current CO hydrogenation process can incorporate captured CO_2_ as a source *via* the optimised reverse water–gas shift reaction, along with low-carbon hydrogen sources.^11^ Another green approach to methanol production is by utilising biomass as feedstock through gasification. Gasification of biomass occurs at high temperatures (800–900 °C) to generate syngas, which is subsequently processed through the shift reaction to achieve the desired hydrogen to CO ratio for methanol production.^20^ Co-electrolysis of CO_2_ and water is another promising technology for producing syngas required for methanol synthesis. Solid oxide electrolysers operating at high temperatures (800–1000 °C) and driven by renewable electricity are used for this purpose.^21^ Despite numerous studies in the literature, significant improvements are still needed to commercialise this process.^22,23^

Previous studies have demonstrated the technical feasibility^24^ and potential climate benefits^25,26^ of green production routes for chemicals. However, for a green pathway to be sustainable, it is also necessary to offer positive economic and social features. Therefore, a common challenge these routes face is their higher production costs compared to fossil-based methods, which makes them nonviable from a sustainability standpoint.^25,27^ Historically, Europe has experienced higher production costs for chemicals due to elevated labour and feedstock expenses.^28^ The global energy market disruption has further amplified this gap. For example, between 2020 and 2022, natural gas prices increased by a factor of 1.6 in the US compared to 9.6 in Europe,^29^ halting more than half of Europe's ammonia production capacity (around 8% of global production^14^) in 2022.^30,31^ As a result, the chemical industry is considering relocating production to regions with lower costs rather than implementing domestic green production strategies. However, this could have detrimental socio-economic effects and undermine Europe's leadership in chemical production, while increasing its susceptibility to supply disruptions. Furthermore, this sector employs approximately 1.2 million people across the continent,^32^ who would be at risk in such an uncertain scenario.

This unexpected situation may present an opportunity to enhance the economic competitiveness, *i.e.*, sustainability of green chemicals, and accelerate the shift to renewable sources. It is yet to be determined whether green chemicals can demonstrate sustainability in addition to their environmental dominance in the current situation. To bridge this crucial research gap, we conduct a monthly techno-economic assessment of Europe's production costs for green and fossil ammonia and methanol.

## Results

### Surging energy prices drive low-carbon technologies

**Fig. 1:**
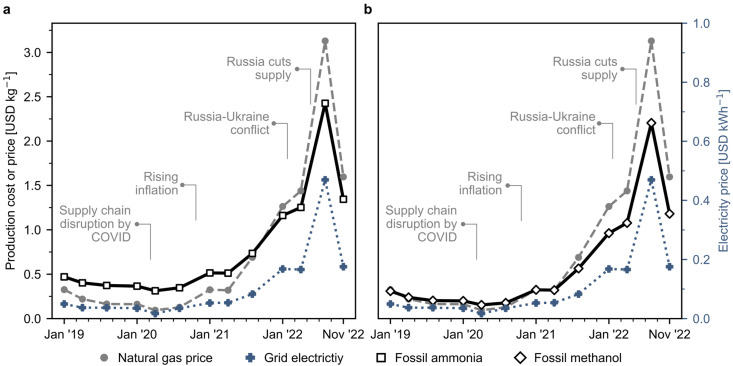
Natural gas and grid electricity spot prices and estimated production costs for Europe's fossil ammonia and methanol. (a) Fossil ammonia produced *via* the Haber–Bosch process with grey hydrogen obtained from natural gas as feedstock. (b) Fossil methanol synthesised through syngas obtained from natural gas. Process simulations contain the necessary information for economic calculations (CAPEX and OPEX). Calculations were performed considering the effect of inflation and the monthly fluctuations in natural gas and electricity prices. The evolution of electricity prices in Europe is individually displayed in Fig. S5.† Monthly analyses for the production costs are available in Fig. S6.†

Fig. S1 and S2† show the basic representation of the ammonia and methanol production process, respectively. Conventionally, ammonia and methanol are produced *via* the steam reforming of natural gas. The detailed process flowsheets are explained in Section 1 of the ESI.† We use natural gas spot prices at the reference Dutch TTF trading hub as a benchmark, acknowledging that ongoing long-term confidential contracts signed by companies may affect actual production costs. Graphical representations for the techno-economic calculations are shown in Fig. S3† for ammonia and Fig. S4† for methanol, respectively. We validated our estimates by comparing them to actual market prices during a stable period before the 2022 disruption, and our results align well with ammonia and methanol market prices over the past few years, as shown in Fig. S7 and S8† respectively. The estimated production cost assuming the use of natural gas sold at current European market prices for fossil ammonia surged from 0.4 USD per kg (averaged value in 2019–2020) to 1.3 USD per kg in November 2022 (Fig. 1a), while fossil methanol costs evolved from 0.2 to 1.2 USD per kg during the same period (Fig. 1b). These increases are directly connected to steadily rising natural gas spot prices due to the COVID-19 pandemic and later to the Russian–Ukraine conflict in February 2022, when volatility increased. Over this unstable period, prices fluctuated considerably and peaked in August 2022 at 2.4 USD per kg for ammonia and 2.2 USD per kg for methanol.

### Cost competitiveness of green ammonia and methanol production routes

We subsequently investigate the economic competitiveness of alternative production routes for ammonia and methanol in Europe, accounting for monthly fluctuations in energy prices. For ammonia, we analysed the Haber–Bosch process using four typical sources of hydrogen:^14^ grey hydrogen from steam methane reforming (SMR), blue hydrogen from SMR coupled with carbon capture and storage (CCS), green hydrogen from water electrolysis using solar photovoltaic (PV) electricity, and green hydrogen from water electrolysis using on-shore wind electricity. Similarly, we compared four alternative production routes for methanol: conventional synthesis from natural gas, synthesis from CO_2_ and blue hydrogen, solar PV-based electrolytic hydrogen, and wind-based electrolytic hydrogen, all using CO_2_ from direct air capture (DAC). Alternatively, it is possible to deploy biomass routes as they are economically appealing and have lower climate change impacts. However, these pathways often lead to burden shifting when considering other environmental impacts over their entire life cycle, such as land use change, resource consumption, and potential impacts on biodiversity and ecosystem.^26^ Section 1 of the ESI† provides more details on production routes.

**Fig. 2:**
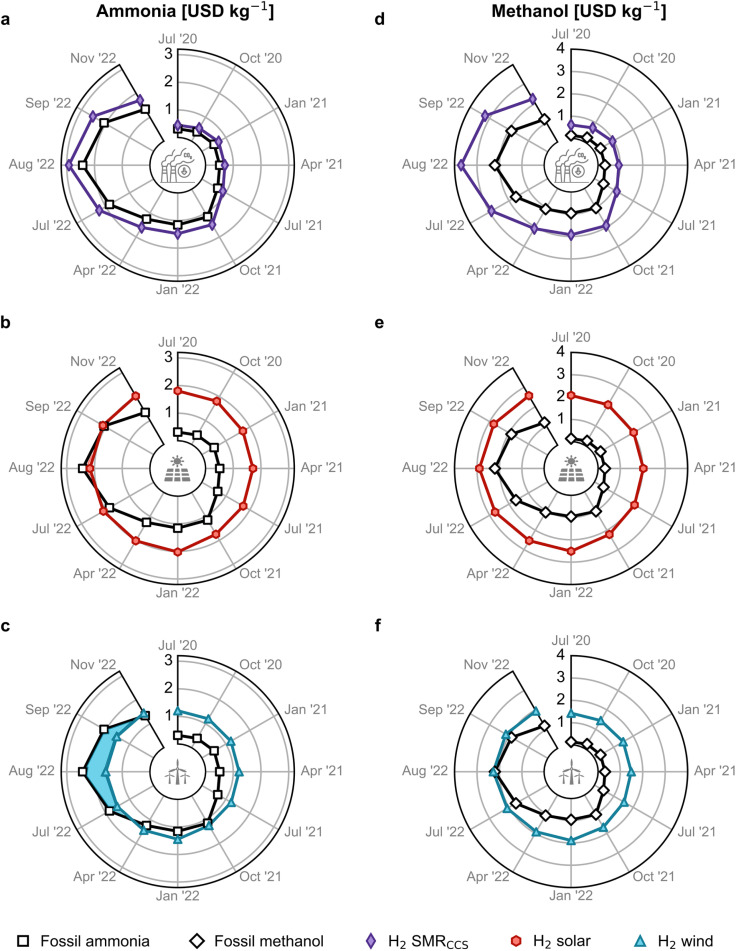
Estimated production cost for green ammonia and methanol in Europe compared with the fossil counterpart. Green ammonia produced *via* the Haber–Bosch process with (a) blue hydrogen from steam methane reforming (SMR) coupled with carbon capture and storage (CCS), green hydrogen from water electrolysis using (b) solar photovoltaic (PV) electricity or (c) on-shore wind electricity. Methanol synthesised from captured CO_2_ and (d) blue hydrogen, (e) PV-based electrolytic hydrogen or (f) wind-based electrolytic hydrogen. Monthly analyses are available in Fig. S13.† The temporal resolution of the period post-July 2022 is enlarged to assess the escalating volatility of fossil routes. A comparison between all the production routes, including electrolysis utilising the grid electricity mix, is shown in Fig. S12.†

Fig. 2 illustrates the cost competitiveness of green *versus* fossil counterparts. As for ammonia, blue hydrogen-based production follows a similar pattern as fossil ammonia, with slightly higher costs due to CCS (Fig. 2a). However, green ammonia produced *via* solar PV-based electrolytic hydrogen has been a close competitor to fossil ammonia prices since matching them in August 2022 (Fig. 2b). Even more remarkably, ammonia produced with hydrogen from water electrolysis using on-shore wind electricity became cheaper from December 2021 to October 2022 at natural gas prices around 1.7 USD per kg (Fig. 1), partly due to the higher capacity factor of wind- *versus* solar-powered water electrolysis (36–39% *versus* 16–18%).^33^ Over this period, wind-based ammonia production costs ranged from 1.3 to 1.6 USD per kg (Fig. 2c, see blue-highlighted area).

The production cost of methanol from DAC CO_2_ and blue hydrogen was 182% higher than fossil methanol on average due to the added expenses of CCS and CO_2_ capture (Fig. 2d). The solar PV-based method was never economically viable (Fig. 2e). However, green methanol generated with wind-based electrolytic hydrogen and DAC CO_2_ reached price parity with fossil methanol in August 2022 at peak natural gas prices of approximately 3.1 USD per kg (Fig. 2f), as the natural gas price strongly affects the total methanol cost, as discussed next.

Fig. S12† provides a graphical comparison of all the ammonia and methanol production routes, including hydrogen from grid electricity-powered water electrolysis. The levelised cost of grid-powered hydrogen is calculated using the levelised cost of electricity shown in Fig. S5.† It is observed that ammonia and methanol from grid electricity have an average production cost of 0.9 USD per kg and 1.2 USD per kg, respectively, from January 2019 to May 2021. These costs are higher than the fossil routes (0.4 and 0.2 USD per kg for ammonia and methanol, respectively) but lower than their corresponding on-shore wind and solar pathways. However, from May 2021, as the grid electricity prices increase in correspondence with the natural gas price, the production cost of grid-based ammonia and methanol becomes economically unfavourable compared to the green routes. Therefore, in this work, we solely focus on the green production routes for ammonia and methanol.

Moreover, all the production cost assessments were performed assuming average European values. However, to assess the national differences, we conducted individual studies for representative countries (Fig. S9 and S10† for ammonia and methanol, respectively). The detailed methodology used to perform these assessments is presented in Section 5 of the ESI.† At the peak of natural gas prices, the production costs of fossil ammonia in Europe ranged from 2.1 to 2.5 USD per kg. Among European countries, Spain displayed costs 13% lower than the European average, while Denmark had the highest prices, 4% higher than the average. Similarly, the production costs for fossil methanol ranged from 2.1–2.2 USD per kg. In 2022, compared to the average production costs in Europe, France recorded the highest prices for ammonia and methanol *via* the wind pathway, with an 11% and 8% increase, respectively. The Netherlands had the highest production costs for the solar pathway, with a 22% increase for ammonia and an 18% increase for methanol. In contrast, Spain demonstrated the lowest production costs for wind-based ammonia, with a 16% reduction, and solar-based ammonia, with a 2% decrease. Moreover, Spain exhibited the most cost-effective wind and solar-based methanol production, with a 3% and 1% reduction, respectively. These prices can be attributed to Spain's reduced levelised electricity costs, up to 35% lower for wind and 22% lower for solar than the European average.

In addition, we conducted a comprehensive analysis of production costs for both the United States (US) and Europe to assess the economic incentives for regions outside Europe to adopt the green transition. The results are presented in Fig. S11.† The US's highest price of fossil ammonia was estimated to be 0.8 USD per kg, nearly 50% lower than the corresponding cost in Europe. Similarly, the fossil route exhibited the highest production cost for methanol at only 0.4 USD per kg, 82% less compared to Europe. These discrepancies can be attributed to the relatively lower prices of natural gas and electricity in the US than in Europe. As a result, the green production routes in the US, despite their environmental advantages, still face substantial economic challenges and remain unsustainable; therefore, the green transition is disincentivised in the US.

**Fig. 3:**
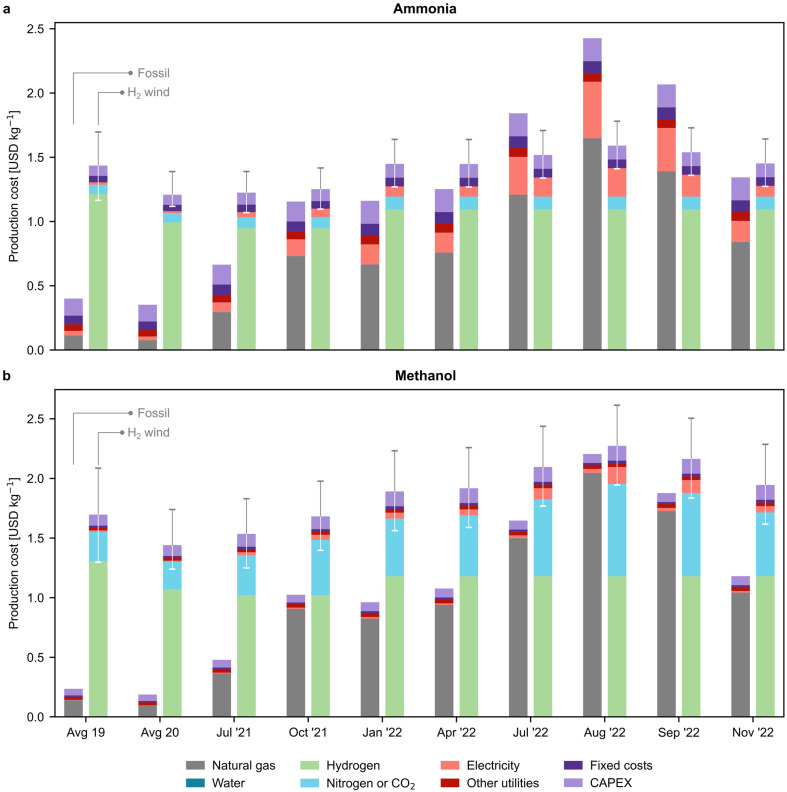
Breakdown of production costs for fossil and green (a) ammonia and (b) methanol. Only the green production routes based on hydrogen from water electrolysis using on-shore wind electricity are displayed. The results for other green routes can be found in Fig. S14.† Error bars indicate the production cost considering the lower and upper bound for the levelised cost of hydrogen (Table S7†). Average values for the years 2019 and 2020 are employed as the natural gas and electricity prices before July 2021 showed negligible variations in comparison.

In Fig. 3, we examine the production cost breakdown of fossil and green chemicals at various time points, with a particular focus on the most promising green routes that rely on wind-based electrolytic hydrogen. The results for other green routes can be found in Fig. S14.† Our findings indicate that the percentage of natural gas in the production cost of fossil ammonia rose from 21% in 2020 to 63% in November 2022 (Fig. 3a). Similarly, for fossil methanol production, its contribution increased from 48% to 88% (Fig. 3b). In contrast, for the green routes, hydrogen represented the highest share of the production cost, accounting for 69–85% for green ammonia and 61–67% for green methanol, depending on the timeframe. On the other hand, our analysis indicates that the average European levelised cost of wind-based electrolytic hydrogen steadily decreased until 2021. However, in 2022, the price of hydrogen experienced a slight increase, which can be attributed to the limited availability of data for the levelised cost of electricity. Specifically, to calculate the levelised cost of hydrogen for 2022, we computed the levelised cost for 2021 and then adjusted it for inflation (as detailed in Section 2.4 of the ESI†). The reduction in on-shore wind electricity costs from 67 to 42 USD per MWh (ref. 33) drove a price decline from 5.2–8.5 USD per kg in 2019 to 5.2–7.3 USD per kg in 2022 after adjusting for inflation. We notice that the hydrogen cost considered in this study aligns well with the lower end of previous literature estimates (*i.e.*, 4.6–10 USD per kg).^34,35^

The cost of the CO_2_ feedstock captured from the atmosphere by DAC is a significant factor in the production cost of green methanol, accounting for up to 28% in November 2022. The rising cost of DAC, from an average of 0.2 USD per kg CO_2_ in 2019 to 0.8 USD per kg CO_2_ in August 2022, is behind this trend. We utilised a high-temperature solvent-based DAC technology, adapted from the work of Keith *etal.*,^36^ which requires around 0.1 kg of natural gas and 77 kWh of electric power per kg of CO_2_ captured, making the capture cost susceptible to fluctuations in natural gas and electricity prices (Fig. S15†).

According to projections, the cost of on-shore wind electricity is expected to reach 40 USD per MWh by 2030.^37^ This is expected to result in a decrease in the hydrogen's levelised cost to approximately 2.8 USD per kg by 2030 and further declining to 2.1 USD per kg by 2050 as described in ref. 38. We performed a sensitivity analysis (Fig. S16†) with varying energy prices to provide a forward-looking perspective. The estimated cost of wind-based ammonia ranges between 0.7 and 0.9 USD per kg, while wind-based methanol is projected to cost between 0.8 to 1.0 USD per kg. Additionally, the breakeven price of natural gas is calculated to be around 0.8–1.1 USD per kg for ammonia and 1.0–1.4 USD per kg for methanol, respectively. Fig. 1 illustrates that the natural gas prices in 2022 were significantly higher than these breakeven prices for most months of the analysis.

### Green ammonia and methanol are sustainable for CO_2_ mitigation

We examine next the estimated avoidance cost, obtained from the production cost and global warming potential (GWP) of green ammonia and methanol in relation to their fossil-based counterparts. For the fossil routes, the GWP impacts are determined to be 2.2 kg CO_2_-eq and 0.7 kg CO_2_-eq per kg of ammonia and methanol, respectively. In contrast, the onshore wind-based ammonia shows a significant reduction in GWP of 68% compared to its fossil counterpart, while the solar-based ammonia achieves a 50% reduction. Similarly, wind-based and solar-based methanol exhibit reductions in emissions of 129% and 37%, respectively.

**Fig. 4:**
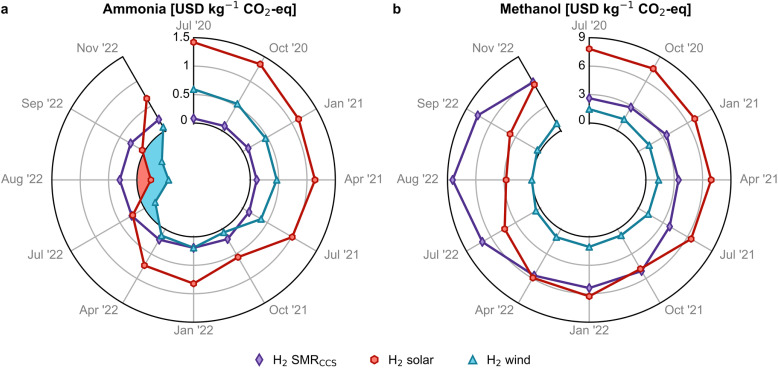
Avoidance costs for green (a) ammonia and (b) methanol production routes. A negative avoidance cost (blue and red shaded region) implies that it is possible to reduce costs and greenhouse gas (GHG) emissions simultaneously. Monthly analyses with error bars are available in Fig. S17.†

In 2020, with an average natural gas price of 0.1 USD per kg, the avoidance cost of wind- and solar-based ammonia was 0.6 and 1.4 USD per kg CO_2_-eq, respectively (Fig. 4a) and much higher for methanol (1.4 and 7.7 USD per kg CO_2_-eq, Fig. 4b), mainly due to the greater monetary gap between fossil and green methanol. The avoidance cost for green ammonia and methanol in 2020 exceeded the removal cost of negative emissions technologies (NETs) such as bioenergy with carbon capture and storage (BECCS) and direct air capture with carbon capture and storage (DACCS).^39^ However, at high natural gas prices in 2022, the avoidance cost of green ammonia became negative, making their use economically attractive for mitigating carbon emissions. A win–win scenario for economic and environmental competitiveness emerged in July 2022 for wind-based ammonia production, which resulted in potential savings of 0.1 to 0.6 USD per kg CO_2_-eq avoided (see blue shaded area in Fig. 4a), making it more sustainable. For methanol *via* wind, a similar scenario emerged in August 2022 with avoidance costs close to zero (Fig. 4b) and savings of 0.3 USD per kg CO_2_-eq avoided (Fig. S17†), considering the lower end of the hydrogen cost range.

## Discussion

In our study, we found that the production cost of fossil ammonia and methanol increased significantly in Europe due to surging energy prices, rising from an average of 0.4 and 0.2 USD per kg in 2019–2020 to 2.4 and 2.2 USD per kg, respectively, during the peak of natural gas spot prices in August 2022. Green ammonia and methanol production became economically attractive during the same period. Notably, wind-based ammonia costs were found to be between 1.3 and 1.6 USD per kg from December 2021 to October 2022 and were more affordable than the fossil route for six months.

Our results indicate that the energy crisis in Europe has had a significant impact on the sustainability level of the low-carbon chemicals industry. Geopolitical instabilities, weather volatility, and increasing competition for liquefied natural gas may continue to hinder a return to the previous pre-crisis situation.^40^ In contrast, advancements in renewable technologies, their widespread implementation, and the positive impact of learning curves are expected to lead to cost reductions.^41^

Europe has committed to reducing its GHG emissions by 55% by 2030 and achieving net zero by 2050, in line with the Paris Climate Agreement.^42^ Significant investment in low-carbon infrastructure is necessary to attain these ambitious targets. However, a recent study indicates that the current rate of investment and development is insufficient to meet these goals.^43^ To successfully reach net zero, an immediate additional investment of approximately 94 billion USD per year until 2025 is necessary. This funding is particularly crucial for renewable power, especially on-shore wind demanding 17.3 billion USD per year, and solar, requiring 7.6 billion USD per year.^43^

Thus, producing green ammonia and methanol through market-driven mechanisms could assist the European chemical industry in reducing GHG emissions at a very low or even negative mitigation cost compared to the use of more expensive and less mature NETs, which may also face, in some cases, specific socio-political barriers.

Both short-term and long-term strategies are required in this situation to accelerate the implementation of green routes. Tailwind regulations are necessary to complement a potentially favourable macroeconomic environment. Additionally, a fraction of the investment should be focused on research and development to enhance current performance and boost emerging technologies with low TRL to address concrete challenges. For both ammonia and methanol production, priority should be given to improving the performance of polymeric membrane water electrolysers to increase competitiveness. Optimised anodes, ideally containing catalysts with lower Pt-group metal content, can achieve technological maturity through lower cell voltages and increased stability.^44,45^ From a broader perspective, the availability of different routes could realise a new scenario where green centralised and decentralised production units coexist. Small electrocatalytic reactors coupled to solar power, known as ammonia leaves,^46^ may open the door to the direct production of fertilisers at croplands. However, direct electrocatalytic reduction of nitrogen is still impractical at an industrial scale, as research must overcome early challenges such as identifying reference catalysts.^46^ As for methanol synthesis, the discovery of catalysts such as promoted indium oxide^47,48^ or zinc–zirconium oxide^49^ has expanded the options for the direct hydrogenation of CO_2_ into methanol beyond traditional copper-based catalysts, whose selectivity and stability could be further improved. However, these new materials have yet to demonstrate increased activity while making further progress in stability and selectivity.^50^ With the development of more active and selective copper electrocatalysts, it may be appealing to synthesise carbon products with direct use as fuels, such as methanol or ethanol, from CO_2_ on a small scale using artificial leaves.^51^ Methanol has remained a minor product in the direct electrocatalytic conversion of CO_2_, though molybdenum-based catalysts have signalled promise for further development.^52^

Our findings indicate that the increasing energy prices in Europe can potentially establish cost-competitive production routes of green ammonia and methanol, thus overcoming the primary obstacle to their implementation in a sustainable chemical industry. By embracing this transformation, the European chemical industry has the opportunity to lead the grand transition in the global movement towards environmentally responsible practices while simultaneously reaping significant economic benefits in the long run. This opportunity becomes even more apparent as fossil resources become scarcer and other geopolitical factors contributing to the market volatility emerge.

## Methods

Section 2 of the ESI† provides a detailed description of the economic assessment methodology, while Section 3 outlines the life cycle activities and environmental assessment methods employed. Additionally, Section 5 elucidates the regionalised economic calculation methodology. This section presents a concise overview of the economic assessments and associated avoidance costs.

### Economic assessment

The production costs of fossil and green ammonia and methanol routes were calculated from the operational and capital expenditures (OPEX and CAPEX). The OPEX term covers both variable expenses such as feedstock and raw materials, as well as fixed expenses including labour, maintenance costs, overhead expenses, and interest. The CAPEX accounts for the equipment cost. The economic assessment calculations are described in detail in Section 2 of the ESI.†

### Avoidance costs

The avoidance cost for each green chemical is as follows:1
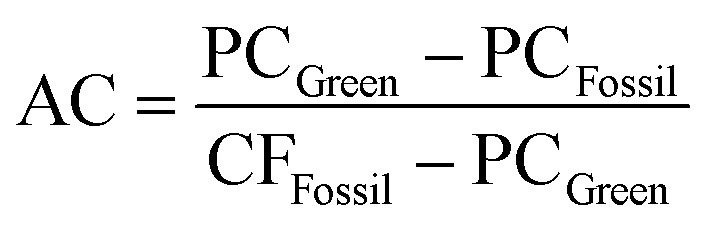
where AC is the avoidance cost in USD per kg CO_2_-eq, PC_Fossil/Green_ represents the production cost in USD per kg for the fossil and green routes, and CF_Fossil/Green_ represents the carbon footprint in kg CO_2_-eq per kg of product.

We estimate the chemicals carbon footprint with an attributional life cycle assessment (LCA) in accordance with the ISO 14040/14044 standards.^53,54^ LCA quantifies environmental impacts over the life cycle of products, from raw materials extraction to end-user disposal.^55^ The functional unit for the LCA is defined as “the production of 1 kg of chemical (ammonia or methanol)”. The scope of the assessment is cradle-to-gate, meaning that the burdens considered cover all the stages from the extraction of raw materials (*e.g.*, natural gas) to the production of the chemicals. The mass and energy flows for the production routes were primarily obtained from D'Angelo *et al.*^25^ and González-Garay *et al.*,^27^ as described above. Data for the background system (*e.g.*, natural gas and electricity supply) were obtained from the Ecoinvent v3.5 database.^56^ Regarding the climate impact assessment, we consider the 100-year global warming potentials (GWPs) as implemented in the ReCiPe 2016 methodology.^57^

## Author contributions

AN: conceptualisation, methodology, visualisation, formal analysis, writing – original draft, writing – review and editing; IRI: conceptualisation, methodology, validation, writing – original draft, writing – review and editing; AM: conceptualisation, methodology, visualisation, writing – original draft, writing – review and editing; JPR: conceptualisation, writing – review and editing, supervision, and project administration; GGG: conceptualisation, validation, writing – original draft, writing – review and editing, supervision, and project administration.

## Conflicts of interest

There are no conflicts to declare.

## Supplementary Material

GC-025-D3GC01053H-s001
