# Supplementary material for: Energy crisis in Europe enhances the sustainability of green chemicals
Source: Green Chem. 2023 Jun 23;25(17):6603–11. doi: 10.1039/d3gc01053h (PMC10464097; doi:10.1039/d3gc01053h)
Supplement: GC-025-D3GC01053H-s001 [file GC-025-D3GC01053H-s001.pdf]

Electronic Supplementary Material (ESI) for Green Chemistry.
This journal is © The Royal Society of Chemistry 2023

Electronic Supplementary Information

Energy crisis in Europe enhances the sustainability of green chemicals

Abhinandan Nabera, Ioan-Robert Istrate, Antonio José Martín, Javier Pérez-Ramírez* and Gonzalo Guillén-Gosálbez*

Institute for Chemical and Bioengineering, Department of Chemistry and Applied Biosciences, ETH Zürich, Vladimir-Prelog-Weg 1, Zürich 8093, Switzerland

1. Production routes

Ammonia

In the work by D'Angelo *et al.*[1], the process models used for the fossil and green ammonia routes were developed in Aspen HYSYS. In order to provide a comprehensive overview of our work, we further outline the process models employed from the original article. For fossil ammonia, natural gas and water are mixed and heated to 858 °C and fed to a steam methane reforming (SMR) unit where natural gas is converted to carbon monoxide and hydrogen (syngas). The output from the SMR is then fed into an autothermal reformer (ATR) along with air (78% nitrogen, 21% oxygen, and 1% argon). The oxygen in the air reacts with the residual methane and increases the temperature to 1,200 °C inside the ATR. The output from the ATR is cooled to 400 °C and sent to a series of water-gas-shift (WGS) reactors to convert carbon monoxide to CO_2. The first one operates at a high temperature (260 °C), *i.e.*, high-temperature water-gas-shift (HTWGS) reactor. The output stream from the HTWGS is fed to a low-temperature water-gas-shift (LTWGS) reactor, which operates at 50 °C. The output stream from the WGS reactors is rich in CO_2, which needs to be removed. This is done in an absorption column using a lean solvent stream with a composition of 90% water and 10% monoethanolamine (MEA) where 99.7% of the input CO_2 is absorbed and removed in the liquid stream. The residual carbon monoxide and CO_2 are eliminated to avoid poisoning the catalyst in the Haber-Bosch process.[2] Accordingly, a methanation unit converts the residual carbon oxides to methane. In the Haber-Bosch process, the CO_2 and carbon monoxide free stream are then compressed to 196 bar, mixed with the recycle stream, and sent to a flash separator. This stream is then sent to a series of three reactors where nitrogen and hydrogen react to produce ammonia. As mentioned previously, the outlet stream from these three reactors is cooled and mixed with the fresh feed and sent to a flash unit. The liquid stream from this flash is rich in ammonia, which is further purified with a pair of flash separators to meet industrial standards (99.6%). For the production of ammonia using green hydrogen (electrolytic hydrogen using wind or solar electricity) and blue hydrogen (SMR coupled with CCS), nitrogen from cryogenic air separation and hydrogen is mixed in the ratio 1:3 and sent to the Haber-Bosch process described earlier. A Proton Exchange Membrane (PEM) electrolyser with a stack lifetime of 60,000 hours is considered for producing green hydrogen *via* water electrolysis. **Figure S1** shows a basic visual representation of the ammonia production process.

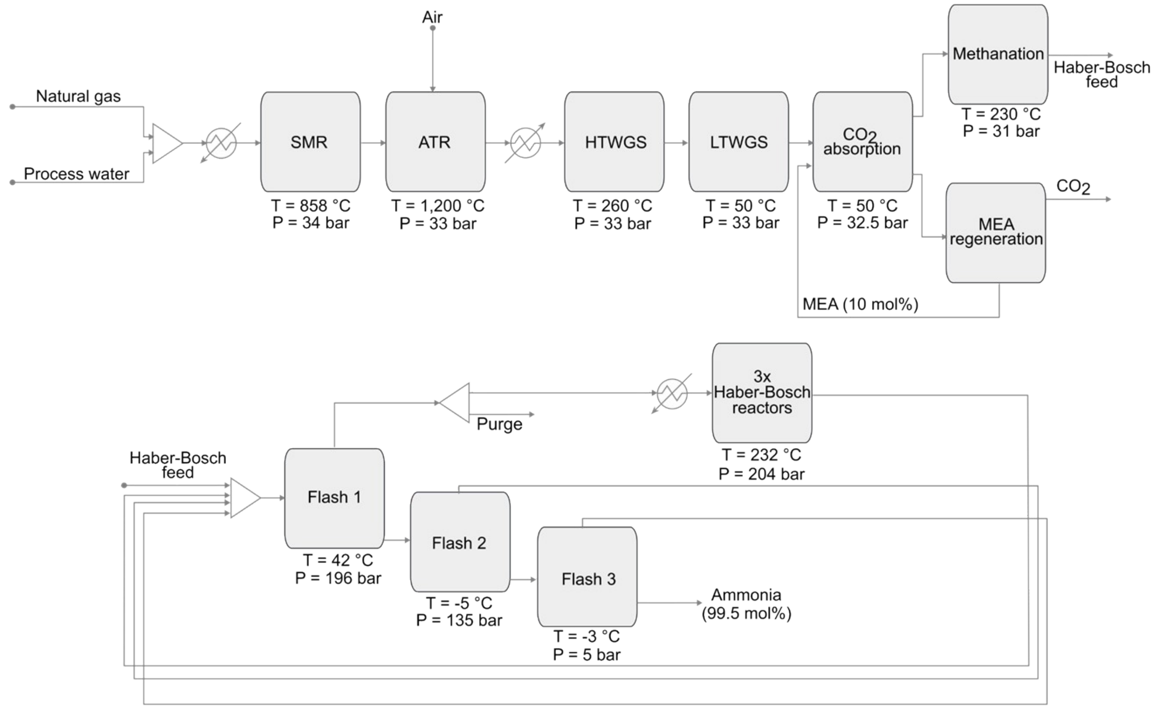

Figure S1. Flowsheet for the production of fossil ammonia. For green ammonia, green hydrogen from water electrolysis and nitrogen from cryogenic air separation are mixed in the ratio 3:1 to prepare the Haber-Bosch feed.[1]

Methanol

The production routes for fossil and green methanol, as outlined in this article, rely on process models originally developed by González-Garay *et al.*[3] using Aspen HYSYS. Fossil methanol is produced from syngas from natural gas *via* SMR. Syngas is then compressed to 110 bar, mixed with the recycle streams and then heated to 150 °C before being fed to a plug-flow reactor where syngas is converted to methanol following the kinetic model in Bussche and Froment.[4] The output stream from the reactor is cooled to 38 °C and sent to a flash separator. A small portion of the gas stream from the flash separator is purged whereas the rest is pressurized to 110 bar and recycled. The liquid stream from the flash separator is expanded to 2 bar and sent to a second flash unit, where the gas stream is again pressurized to 110 bar and recycled, and the liquid stream is sent to a distillation column. The distillation column separates liquid methanol (at the condenser) with a purity of 99.9% and water (at the bottom). To produce methanol using green and blue hydrogen, CO_2 from DAC and hydrogen is mixed with the recycle streams, compressed to 50 bar, and heated to 180-280 °C. This stream is then sent to a reactor where, the reverse water-gas shift (RWGS) reaction takes place, converting CO_2 to carbon monoxide, which is consequently hydrogenated to methanol. Similar to fossil methanol, the outlet from the reactor is sent to a series of two flash separators where the gas stream from the first flash is recycled (with a purge). The liquid stream from the second flash unit is sent to a distillation column which recovers methanol at a purity of 99.9%. The CO_2 fed to the reactor is obtained *via* the DAC technology adapted from the work by Keith *et al.*[5] Here, aqueous potassium hydroxide (KOH) is used as a sorbent and a caustic calcium cycle is implemented for the regeneration of KOH. However, energy from natural gas is required for the calciner in the recovery cycle. **Figure S2** shows a basic visual representation of the methanol production process.

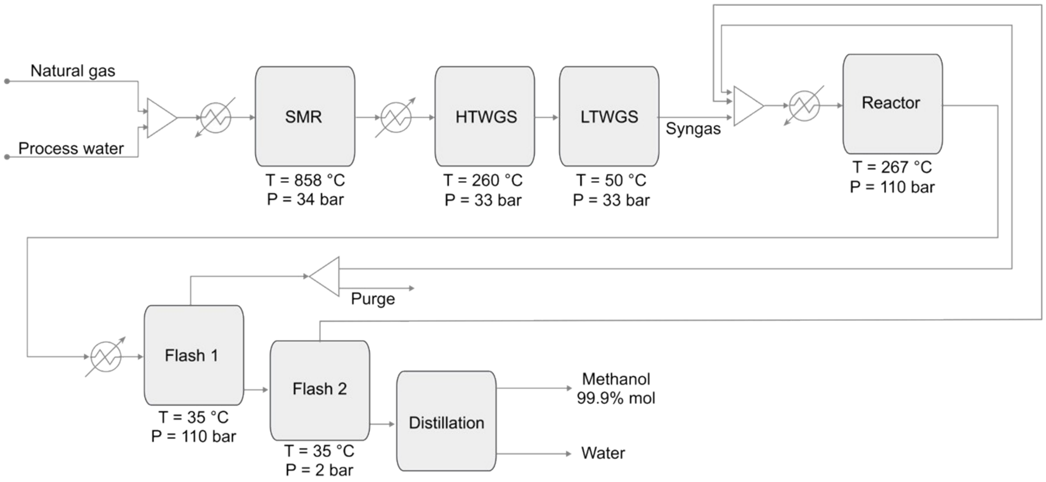

Figure S2. Flowsheet for the production of fossil methanol. For green methanol, CO_2 from DAC and hydrogen from water electrolysis are mixed in the ratio of around 1:3 and the reactor operates at 50 bar.[3]

2. Economic assessment

We used data from process simulations to determine the energy and material inputs and outputs as well as the necessary infrastructure associated with each production route. We then estimated the operational and capital expenditures (OPEX and CAPEX) for a reference year (2019), adjusting afterwards the results for other years using the Chemical Engineering Plant Cost Index (CEPCI) to account for inflation. For the OPEX calculations, the natural gas and electricity prices were not adjusted for inflation but instead we considered explicitly its fluctuating price at any given point in time (monthly average) based on historical market values. The hydrogen cost, which is part of the OPEX, was estimated from the renewable electricity price and the electrolyser cost following the method in Section 2.4. **Figure S3** and **Figure S4** show a graphic representation of the economic models for the estimation of production costs of fossil and green ammonia and methanol, respectively. The data used is presented in the following sections.

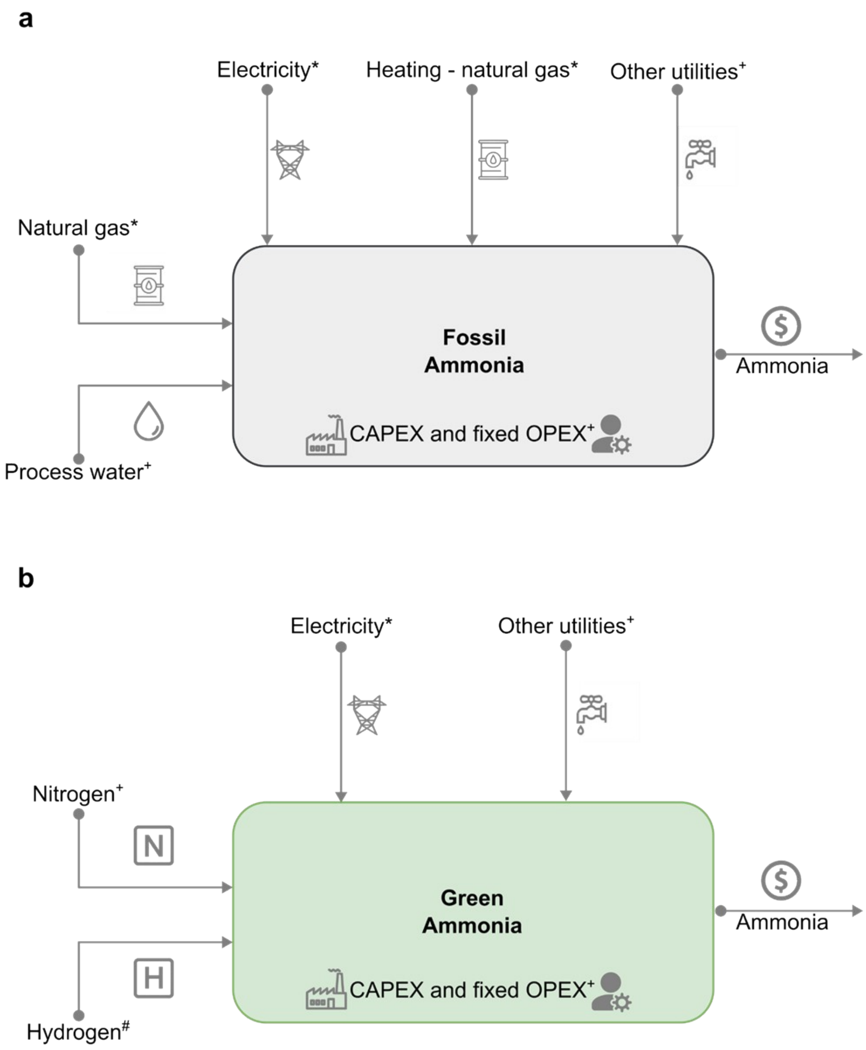

*monthly average prices (Figure S6); +updated for inflation via CEPCI (Table S3); #LCOH from Table S7

Figure S3. Techno-economic model for the estimation of production costs for (a) fossil ammonia and (b) green ammonia. Other utilities include cooling water and wastewater treatment.

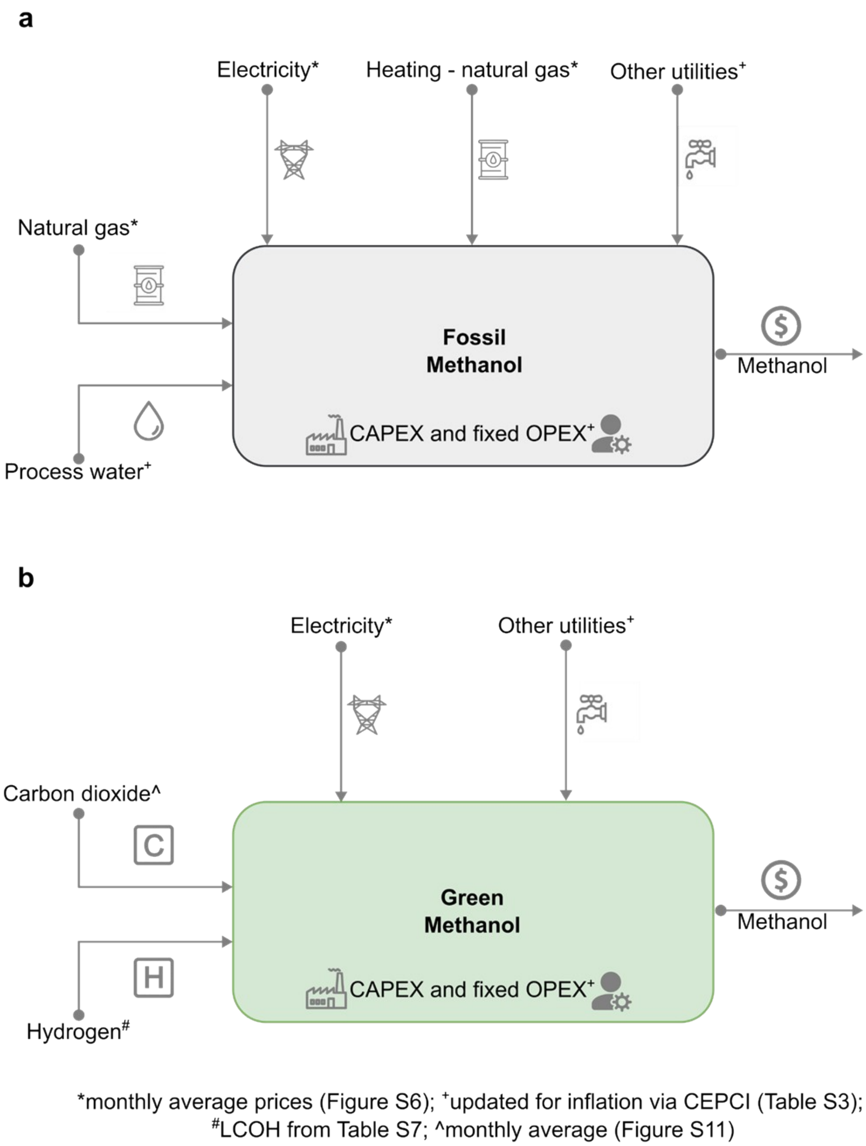

Figure S4. Techno-economic model for the estimation of production costs for (a) fossil methanol and (b) green methanol. Other utilities include cooling water and wastewater treatment.

2.1. Energy and materials inputs and outputs

The mass and energy balances for both fossil and green ammonia are taken from process models developed in the original work by D'Angelo *et al*.[1] and shown in **Table S1**. The inventories for fossil and green methanol are similarly retrieved from process models employed in the work by González-Garay *et al*.[3] and are shown in **Table S2**.

Table S1. Inputs and outputs of the ammonia production routes. These values are extracted from the work done by D'Angelo *et al.*[1]

Material/energy stream	Fossil route	Green route
**Feedstock**		
Natural gas [kg]	0.43	-
Process water [kg]	0.83	-
Nitrogen [kg]	-	0.82
Hydrogen [kg]	-	0.18
**Utilities**		
Heating – natural gas [kg]	0.10	-
Electricity [kWh]	935.39	469.89
Cooling water [GJ]	18.12	3.12
CO_2 emissions [kg]	1.43	-

Table S2. Inputs and outputs of the methanol production routes. These values are extracted from the work done by González-Garay *et al.*[3]

Material/energy stream	Fossil route	Green route
**Feedstock**		
Natural gas [kg]	0.51	-
Process water [kg]	0.85	-
CO_2 feedstock [kg]	-	1.45
Hydrogen [kg]	-	0.19
**Utilities**		
Heating – natural gas [kg]	0.15	-
Electricity [kWh]	74	300
Cooling water [GJ]	-	4.95

2.2. Data for CAPEX calculations

The OPEX were determined from the mass and energy flows retrieved from the process models and applying the corresponding cost factors, while the CAPEX was directly taken from the original sources. Specifically, the CAPEX contributions for fossil and green ammonia were taken from D'Angelo *et al.*[1] With 2019 as the reference year, the contribution of CAPEX is estimated to be around 133 USD t^{-1} and 80 USD t^{-1} of ammonia for the fossil and green routes, respectively. Similarly, for the case of fossil and green methanol, the CAPEX is based on the process models developed by González-Garay *et al.*[3] For 2019, the CAPEX contribution stood at around 56 USD t^{-1} and 92 USD t^{-1} of methanol for the fossil and green routes, respectively. In the original references[1,3], the total CAPEX for fossil and green ammonia and methanol were calculated using the correlations from Sinnot and Towler[6] incorporating the installation factors for each equipment used. Next, equation (9.27) from Sinnot and Towler was utilised to annualise the total CAPEX.[6] All costs have been converted to $USD_{2020\text{-}22}$ using the CEPCI (**Table S3**) while considering different operating years other than 2019.

Table S3. Chemical Engineering Plant Cost Index (CEPCI) from 2019 to 2022. Due to lack of availability of data, an average from January 2022 to September 2022 is considered as the CEPCI for 2022.

Year	CEPCI
2019	607.5
2020	596.2
2021	708.0
2022 (average till September)	817.5

2.3. Data for OPEX calculations

The prices of feedstock and utilities other than natural gas, electricity, CO_2 feedstock, hydrogen are based on D'Angelo *et al.*[1] (**Table S4**). These prices are adjusted for inflation using the CEPCI when considering data for a different year.

Table S4. **Cost parameters used in the OPEX calculations.** All costs are presented in USD_{2019}. These costs are further converted to $USD_{2020\text{-}22}$ using the CEPCI.

Flow	Value
**Ammonia**	
Water [USD t^{-1}]	0.18
Cooling water [USD GJ^{-1}]	0.38
CO_2 emissions [USD t^{-1}]	30
Nitrogen [USD t^{-1}]	91
**Methanol**	
Steam [USD t^{-1}]	15.6
Cooling water [USD m^{-3}]	0.03
Catalyst [USD t^{-1}]	136
Wastewater treatment [USD m^{-3}]	1.64

For natural gas, we consider the monthly average spot prices at the Title Transfer Facility (TTF) hub in the Netherlands as reported in the Commodity Market Outlook of the World Bank (see **Figure 1** in the main manuscript).[7] The Dutch TTF hub has gained significant importance due to its high trade volumes, and currently acts as an European and global benchmark for natural gas prices.[8] Moreover, we consider monthly average electricity prices as shown in **Figure S5**.[9]

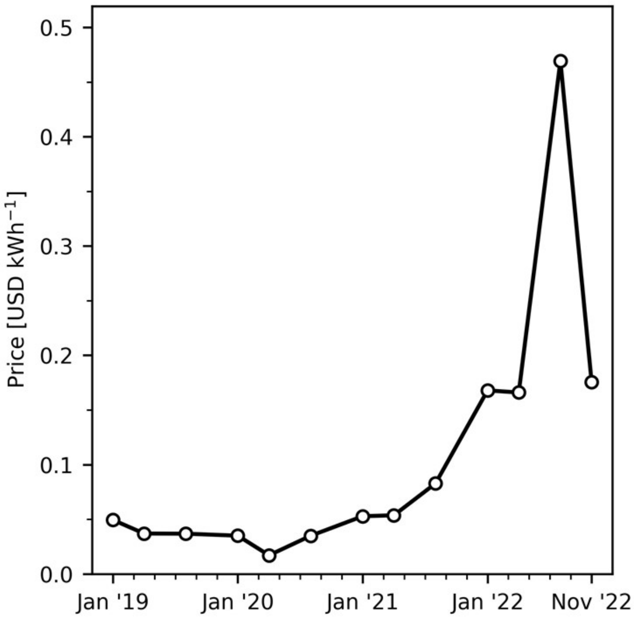

Figure S5. Evolution of monthly average electricity prices in Europe. Notably, electricity costs per kWh increased steadily since July 2021 following similar reasons as discussed in the case of natural gas (see **Figure 1** in the main manuscript). The monthly average prices of electricity along with natural gas are used in the techno-economic models for calculating the production cost estimates.

Figure S6 shows the monthly average natural gas spot prices in addition to the fossil ammonia and methanol production cost. Calculations were performed considering the effect of inflation and the monthly fluctuations in natural gas and electricity prices (the evolution of electricity prices in Europe is displayed in **Figure S5**). Notably, ammonia and methanol production costs have increased steadily since 2020, due to the rising inflation as a consequence of the COVID-19 pandemic, with a sharp increase since February 2022, after the Russian invasion of Ukraine.

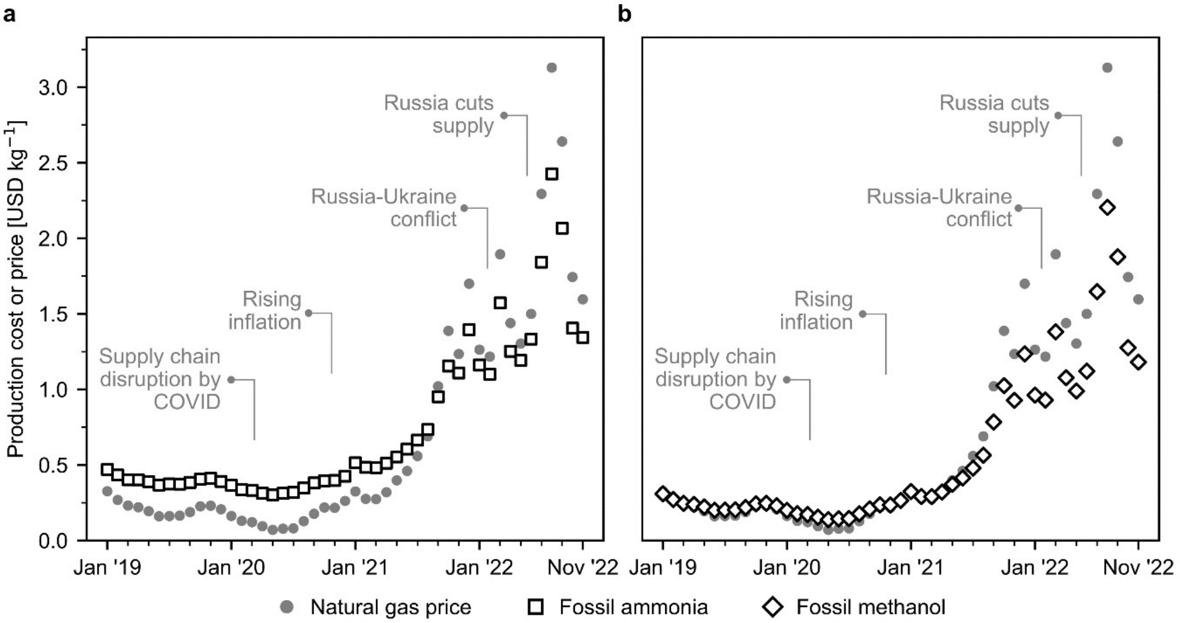

Figure S6. Natural gas spot prices and production cost for fossil ammonia and methanol in Europe. (a) Fossil ammonia produced *via* the Haber-Bosch process with grey hydrogen from steam methane reforming (SMR) using natural gas as feedstock. **(b)** Fossil methanol synthesised from natural gas.

The cost of CO_2 from Direct Air Capture (DAC) is taken from Keith *et al.*[5] and updated with the new natural gas and electricity prices, thus obtaining the monthly cost shown in **Figure S15**. This is done by subtracting the cost of these two energy inputs at the prices in the original reference, and adding the updated values at the prices considered in this work. It should be noted that the price of CO_2 capture is highly influenced by the natural gas and electricity prices as around 0.1 kg of natural gas and 77 kWh of electricity is required per kg of CO_2 captured.

2.4. Data for levelised cost of hydrogen (LCOH) calculations

The levelised cost of hydrogen (LCOH) is calculated as in D'Angelo *et al.*[1] A Proton Exchange Membrane (PEM) electrolyser with a stack lifetime of 60,000 hours is considered for the analysis.[1] The parameters used to calculate the LCOH are shown in **Table S5**.

Table S5. Parameters for the Proton Exchange Membrane (PEM) electrolyser.[1] All values are in reference to the year 2019. These values are used to calculate the levelised cost of hydrogen using the methodology explained further.

**Component**	**Value**
Nominal input power [kW]	5,000
Nominal hydrogen flow [kg h^{-1}]	90
Stack lifetime [h]	60,000
Power demand electrolyser [kWh Nm^{-3} H_2]	5
Capital cost [USD kW^{-1}]	1,124
Interest rate [%]	6.4
Other costs factor (fraction of initial CAPEX)	0.04
Decommissioning factor (fraction of depreciable capital)	0.10
Equipment replacement factor (fraction of initial CAPEX)	0.15
Construction period [yr]	2
Start-up period [yr]	1
Start-capacity [%]	50
Capital investment allocation [%]	50
Annual operation [h]	8,760

First, we calculate the life span of the PEM electrolyser using the stack lifetime and the capacity factors for the specific technology (on-shore wind or solar PV) from **Table S6** (**Equation S1**).

$$\text{Life span [yr]} = \frac{\text{stack lifetime} \cdot 100}{\text{annual hours of operation} \cdot \text{capacity factor}} \quad \textbf{(Equation S1)}$$

The amount of hydrogen produced annually is then calculated assuming a nominal hydrogen flow per stack using **Equation S2**.

$$\text{Annual } H_2 \text{ production [kg yr}^{-1}] = \frac{\text{nominal } H_2 \text{ flow per stack} \cdot \text{stack lifetime}}{\text{life span}} \quad \textbf{(Equation S2)}$$

Assuming a capital investment allocation of 50% for two years with one year of construction completed[1], the CAPEX is estimated using the capital cost and nominal input power employing **Equation S3**. Notably, the capital cost is with reference to the year 2019 and is updated for inflation using the CEPCI when considering data for a different year.

$$\text{CAPEX [USD]} = \sum_{i=-1}^{0} \frac{\text{nominal input power} \cdot \text{capital cost} \cdot \frac{\text{capital investment allocation}}{100}}{(1 + \text{interest rate})^{i}} \quad \textbf{(Equation S3)}$$

The amount of hydrogen produced throughout the life span of the electrolyser is then calculated using **Equation S4**. We assume that in the first year of operation the plant works at a 50% capacity.[1] Therefore, only for the first year, the hydrogen produced is multiplied by a factor of 0.5.

$$H_2 \text{ produced [kg]} = \sum_{i=1}^{\text{life span}} \text{annual } H_2 \text{ production} \cdot H_2 \text{ production capacity}(i)$$

$$\text{with } H_2 \text{ production capacity}(i) = \begin{cases} 0.5 & i = 1 \\ 1.0 & i > 1 \end{cases} \quad \textbf{(Equation S4)}$$

In the next step, the total OPEX are calculated by summing the electricity costs (**Equation S5**) using the levelised cost of electricity (LCOE), other costs as 4% of initial CAPEX (**Equation S6**), plant decommissioning as 10% of initial CAPEX (**Equation S7**), and equipment replacement costs as 15% of depreciable CAPEX (**Equation S8**).[1,10]

$$\text{Electricity cost [USD]} = \sum_{i=1}^{\text{life span}} \frac{\text{power demand electrolyser} \cdot \text{LCOE} \cdot \text{H}_2 \text{ produced}}{(1+\text{interest rate})^i} \quad \textbf{(Equation S5)}$$

$$\text{Other costs [USD]} = \sum_{i=1}^{\text{life span}} \frac{\text{nominal input power} \cdot \text{capital cost} \cdot \text{other costs factor}}{(1+\text{interest rate})^i} \quad \textbf{(Equation S6)}$$

$$\text{Plant decommissioning [USD]} = \sum_{i=1}^{\text{life span}} \frac{\text{nominal input power} \cdot \text{capital cost} \cdot \text{decommissioning factor}}{(1+\text{interest rate})^i} \quad \textbf{(Equation S7)}$$

$$\text{Equipment replacement costs [USD]} = \sum_{i=1}^{\text{floor}(\frac{\text{life span}}{7})} \frac{\text{nominal input power} \cdot \text{capital cost} \cdot \text{equipment replacement factor}}{(1+\text{interest rate})^i} \quad \textbf{(Equation S8)}$$

$$\text{OPEX [USD]} = \text{electricity cost} + \text{other costs} + \text{plant decommissioning} + \text{equipment replacement costs} \quad \textbf{(Equation S9)}$$

We retrieved European average LCOE for on-shore wind and solar electricity and their corresponding capacity factors from the International Renewable Energy Agency (IRENA)[11] for 2019 to 2021, as shown in **Table S6**.

Table S6. Levelised cost of electricity (LCOE) in USD MWh^{-1} and capacity factors (CF) in % for Europe.

**Power technology**	**2019**				**2020**				**2021**			
	**Avg.**	**Low**	**High**	**CF**	**Avg.**	**Low**	**High**	**CF**	**Avg.**	**Low**	**High**	**CF**
Wind - on shore	67	37	96	35.6	45	35	65	36.0	42	26	59	39.0
Solar PV	68	52	190	17.5	57	39	163	16.1	48	29	120	17.2

In the final step, the LCOH is calculated by summing the CAPEX and OPEX and dividing by the total amount of hydrogen produced as shown in **Equation S10**.

$$\text{LCOH [USD kg}^{-1}\text{ H}_2\text{]} = \frac{\text{CAPEX} + \text{OPEX}}{\text{H}_2 \text{ produced} \cdot \sum_{i=1}^{\text{life span}} \frac{1}{(1+\text{interest rate})^i}} \quad \textbf{(Equation S10)}$$

Due to data gaps for 2022, we determine first the LCOH for 2021 and then adjust it for inflation using the CEPCI. The calculated average, low, and high LCOH for each power technology in each year is reported in **Table S7**.

Table S7. Levelised cost of hydrogen (LCOH) in USD kg^{-1}. Section 2.4 provides a comprehensive explanation of the assumptions and methodology employed for computing the LCOH values.

Power technology	2019			2020			2021			2022		
	**Avg.**	**Low**	**High**	**Avg.**	**Low**	**High**	**Avg.**	**Low**	**High**	**Avg.**	**Low**	**High**
Wind - on shore	6.88	5.23	8.48	5.64	5.09	6.74	5.38	4.50	6.31	6.21	5.19	7.29
Solar PV	9.22	8.34	15.9	9.05	8.06	14.88	8.21	7.17	12.17	9.48	8.28	14.06

2.5. Total cost calculations

The total costs for the fossil routes were calculated as the summation of the CAPEX and OPEX values estimated in Sections 2.2 and 2.3. Production volumes were extracted from the original references and then used to calculate the production cost per kg of ammonia or methanol.[1,3] Similar steps were followed for the green routes. However, for the OPEX calculations, the electrolytic hydrogen costs were calculated using the methodology outlined in Section 2.4. These costs were then combined with the OPEX calculations from Section 2.3 to determine the total OPEX.

3. Carbon footprint assessment

The activities used from the Ecoinvent v3.5[12] database to calculate the carbon footprint are shown in **Table S8**.

Table S8. Inventories used from Ecoinvent v3.5 database[12] for the background system. These activities are used to calculate the global warming potential and the avoidance costs for the different routes of production of ammonia and methanol.

Flow	Output	Activity in Ecoinvent v3.5
Natural gas	Fossil ammonia and methanol, CO_2 from DAC, H_2 *via* SMR + CCS	natural gas production [RoW]
Process water	Fossil ammonia and methanol, CO_2 from DAC	tap water production, conventional treatment [RoW]
Grid electricity	All scenarios	market group for electricity, high voltage [GLO]
Air separation facility	Nitrogen	air separation facility construction [RoW]
Cooling water	All scenarios	water, cooling, unspecified natural origin [RoW]
Solar electricity for water electrolysis	H_2 *via* solar	electricity production, photovoltaic, 570kWp open ground installation, multi-Si [RoW]
Wind electricity for water electrolysis	H_2 *via* wind	electricity production, wind, >3MW turbine, onshore [RoW]
Calcium carbonate for DAC technology	CO_2 from DAC	market for calcium carbonate, precipitated [RER]
Heat from steam	Non-fossil methanol	market for heat, from steam, in chemical industry [RER]
Wastewater treatment	Non-fossil methanol	market for wastewater, average [RoW]

4. Model validation

To validate the estimated production costs for fossil ammonia and methanol, we used their respective market prices and computed Pearson correlation coefficients (r) and the mean absolute percentage errors (MAPE). The production cost estimates for ammonia calculated *via* techno-economic models were compared with the data extracted from Green Markets® (**Figure S7**). Green Markets® has recently published the market price of fossil ammonia from July 2019 to July 2022 for Western Europe. **Figure S7a** show the evolution of Europe's market price and production cost (estimated in this work). In **Figure S7b**, we compare the two data series using the Pearson correlation coefficient (r) and the mean absolute percentage error (MAPE). The r value is found to be around 0.95 showing strong correlation between the two data series. Similarly, the MAPE is found to be around 29.7% for the time period January 2019 to December 2020 and 37.7% for the time period January 2019 to November 2022. Notably, here the error is calculated between the market price and the estimated production cost.

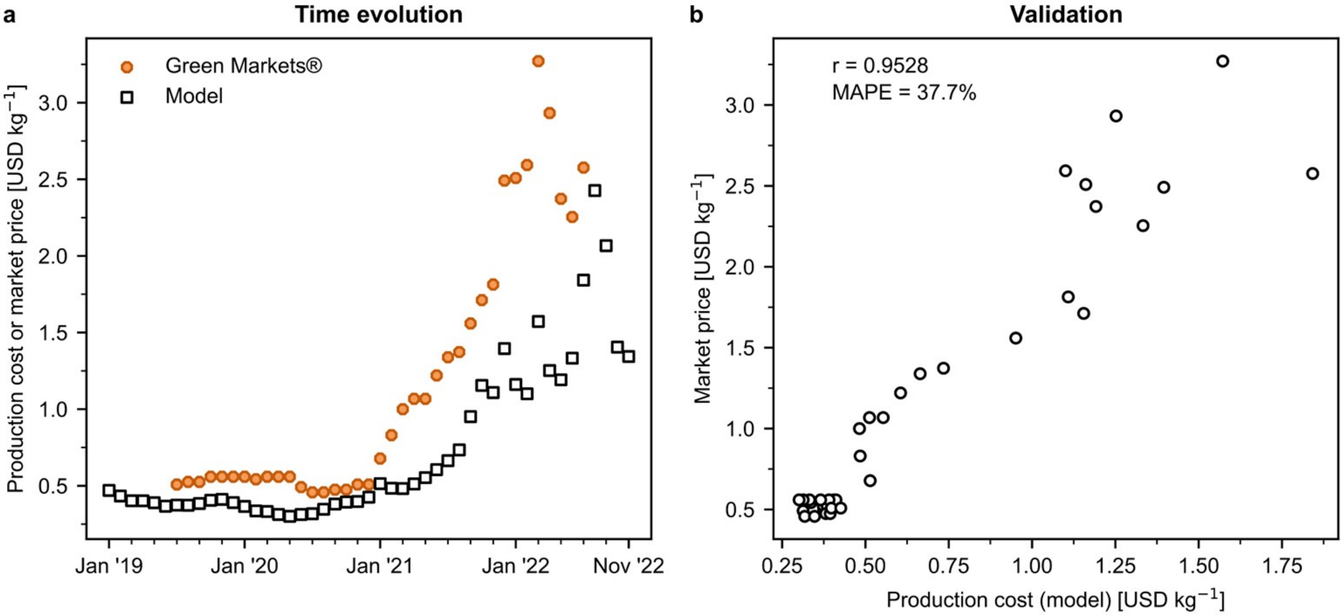

Figure S7. Ammonia model validation. (a) time evolution of the production cost estimates in comparison to the market price extracted from Green Markets® and **(b)** correlation between the market price and the production cost estimate. A Pearson correlation coefficient (r) value of 0.95 is estimated showing a strong correlation between the two data series. Similarly, a mean absolute percentage error (MAPE) of 38% is calculated between the market price and the estimated production cost.

Market prices for European methanol were retrieved from Methanex Corporation (January 2015 to November 2022). Similar to ammonia, the data from Methanex is used to validate our production cost estimates from January 2015 to November 2022. The evolution of Europe's market price and estimated production cost is depicted in **Figure S8a**. The r value and the MAPE from January 2015 to July 2021 is calculated to be around 0.75 and 48.6%, respectively (**Figure S8b**) showing a high correlation between the two data series. For the entire time range (January 2015 to November 2022), the r coefficient decreases to 0.68 and the MAPE increases to 49.2%. Also, there is a visible difference between the market price and estimated production cost for fossil methanol from July 2021. This difference is thought to exist due to several reasons. On the one hand, Methanex has production sites in countries not affected by the current volatility of natural gas prices and hence produce methanol at a lower cost. When this methanol is exported to Europe, the market price is expected to be lower than the cost of production in Europe. On the other hand, we use monthly average natural gas spot prices from the TTF trading

hub in the Netherlands. Companies with ongoing long-term contracts may secure a more favourable gas price compared to the current spot price, and the production cost would be lower as well. However, we would expect that the price of natural gas agreed upon in deals closed since the outbreak of the energy crisis would depend on the natural gas TTF index. Therefore, our estimates do not correspond to actual market values, but to the minimum selling prices in the market if those chemicals were produced in EU at the corresponding natural gas prices.

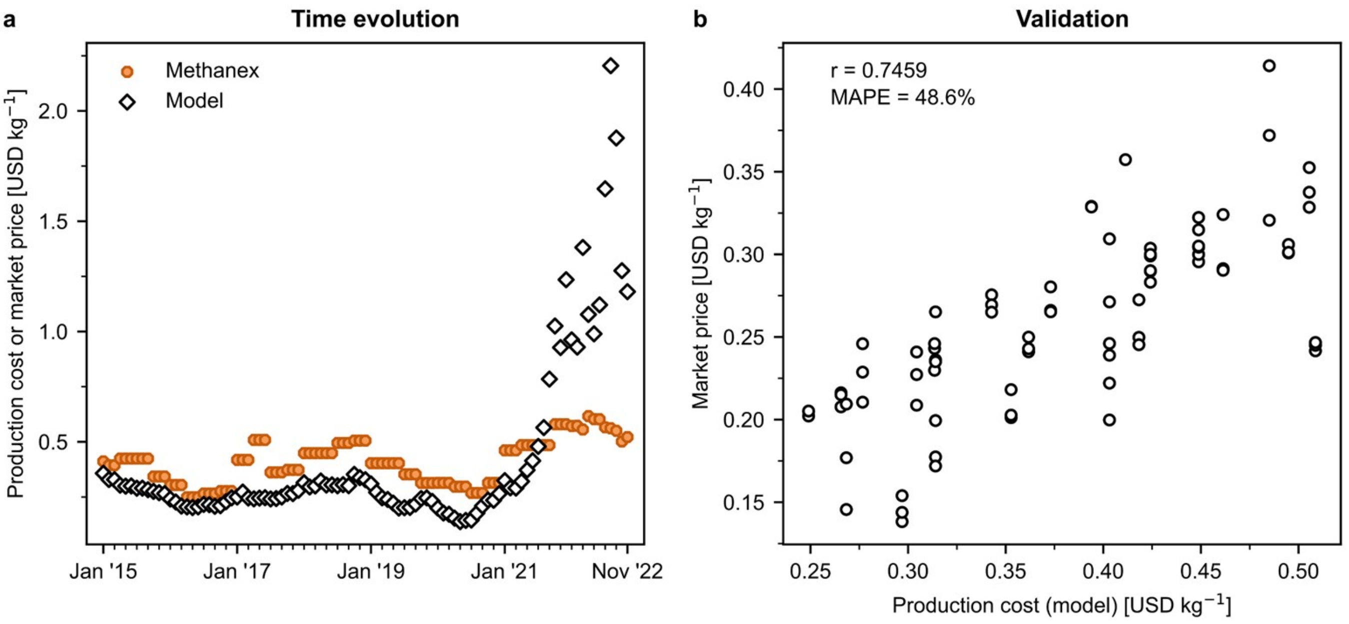

Figure S8. Methanol model validation. (a) time evolution of the production cost estimates in comparison to the market price extracted from Methanex and **(b)** correlation between the market price and the production cost estimate. A Pearson correlation coefficient (r) value of 0.75 is estimated for the time period January 2015 to July 2021 (shown) and 0.68 for the time period January 2015 to November 2022 (not shown). Similarly, an MAPE of 48.6% is estimated for the time period January 2015 to July 2021.

5. Regional cost analysis

In our primary analysis, we utilise average prices for Europe to determine the production costs. However, to assess the viability of these calculations, we conduct region-wise calculations to determine the production costs in various regions within Europe. The methodology for these calculations is explained in detail in Section 5.1. Additionally, in Section 5.2, we compare the production cost estimates of Europe with those of the United States (US). **Table S9** and **Table S10** display the levelised cost of electricity and hydrogen for the different regions considered in this analysis, specifically for wind and solar, respectively. We retrieved regional LCOE for on-shore wind and solar electricity and their corresponding capacity factors from the International Renewable Energy Agency (IRENA)[11] for 2019 to 2021.

Table S9. Region-wise levelised cost of electricity (LCOE) in USD MWh^{-1}, levelised cost of hydrogen (LCOH) in USD kg^{-1} and capacity factors (CFs) in % for solar power. Due to lack of available data for the capacity factors, the global average was assumed for all the regions. The global average capacity factors are 17.5% for 2019, 16.1% for 2020, and 17.2% for 2021.

Country	2019			2020			2021			2022
	LCOE	CF	LCOH	LCOE	CF	LCOH	LCOE	CF	LCOH	LCOH
Germany	88	17.5	10.32	72	16.1	9.86	65	17.2	9.12	10.53
France	82	17.5	10.01	72	16.1	9.88	52	17.2	8.41	9.71
Italy	59	17.5	8.71	62	16.1	9.29	52	17.2	8.41	9.71
Turkey	78	17.5	9.76	60	16.1	9.18	65	17.2	9.11	10.53
Spain	57	17.5	8.62	44	16.1	8.35	48	17.2	8.23	9.50
Netherlands	128	17.5	12.51	108	16.1	9.86	65	17.2	9.12	10.53
United States	68	17.5	9.09	57	16.1	9.08	55	17.2	8.64	9.97

Table S10. Region-wise levelised cost of electricity (LCOE) in USD MWh^{-1}, levelised cost of hydrogen (LCOH) in USD kg^{-1} and capacity factors (CFs) in % for wind power.

Country	2019			2020			2021			2022
	LCOE	CF	LCOH	LCOE	CF	LCOH	LCOE	CF	LCOH	LCOH
Denmark	64	39	6.58	48	39	5.71	47	39	5.63	6.50
Germany	69	31	7.36	50	34	6.03	40	28	5.97	6.90
France	65	33	6.97	48	32	6.13	51	36	5.98	6.90
Sweden	46	38	5.59	40	38	5.27	36	37	5.15	5.95
Italy	66	33	7.00	54	33	6.34	42	33	5.68	6.56
Turkey	66	34	6.91	42	37	5.46	50	39	5.82	6.72
Spain	67	39	6.75	42	38	5.36	27	43	4.46	5.15
United States	51	44	5.69	37	43	4.99	31	45	4.53	5.23

5.1. Europe

Figure S9 illustrates the regional assessment of production costs for ammonia in various European countries, while **Figure S10** presents the regional assessment for methanol. To calculate the production costs of fossil ammonia and methanol, we employ a methodology similar to the one described in Section 2. The only variable we consider is the regional grid electricity costs for our calculations. Furthermore, the fixed costs are multiplied by the Purchase Power Parity (PPP) ratio specific to each country in relation to Europe. Similarly, the prices were calculated for the green routes using the methodology specified in Section 2, considering the LCOE and their corresponding capacity factors represented in **Table S9** and **Table S10** for solar and wind, respectively. The LCOE and capacity factors were further utilised to calculate the LCOH, as described in Section 2.4. Due to data gaps for 2022, the LCOH was initially determined for 2021 and then adjusted for inflation using the CEPCI. The calculated LCOH for each region in each year is reported in **Table S9** for solar and **Table S10** for wind, respectively.

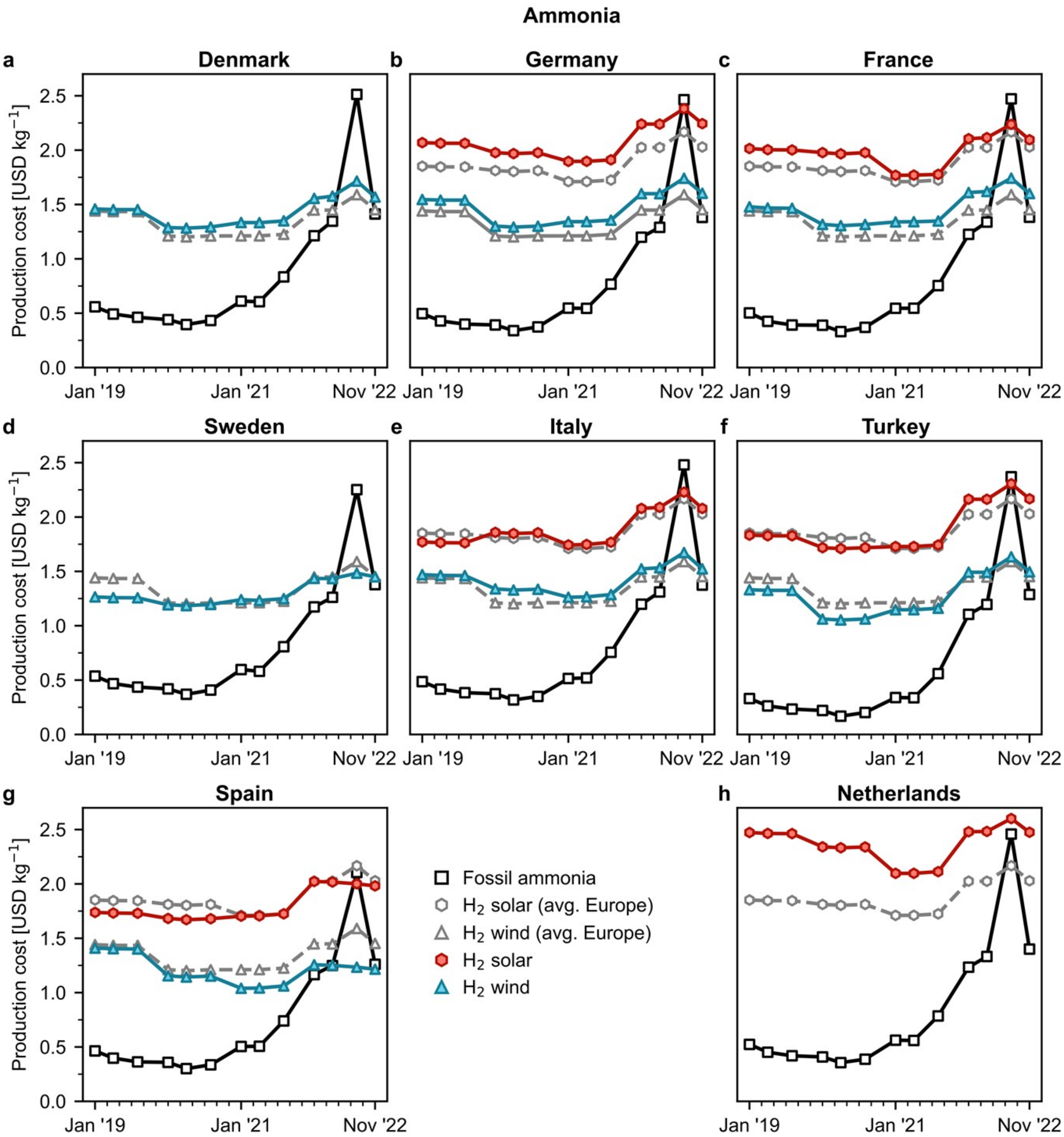

Figure S9. European regional production costs for fossil and green ammonia in (a) Denmark, (b) Germany, (c) France, (d) Sweden, (e) Italy, (f) Turkey, (g) Spain, and (h) Netherlands. The levelised cost of electricity and hydrogen used to calculate the production cost are shown in **Table S9** for solar and **Table S10** for wind, respectively. Access to solar data for Denmark and Sweden and wind data for the Netherlands was unavailable. The fossil cost estimates are based on the Dutch TTF index for natural gas spot prices, as shown in **Figure 1** of the main manuscript.

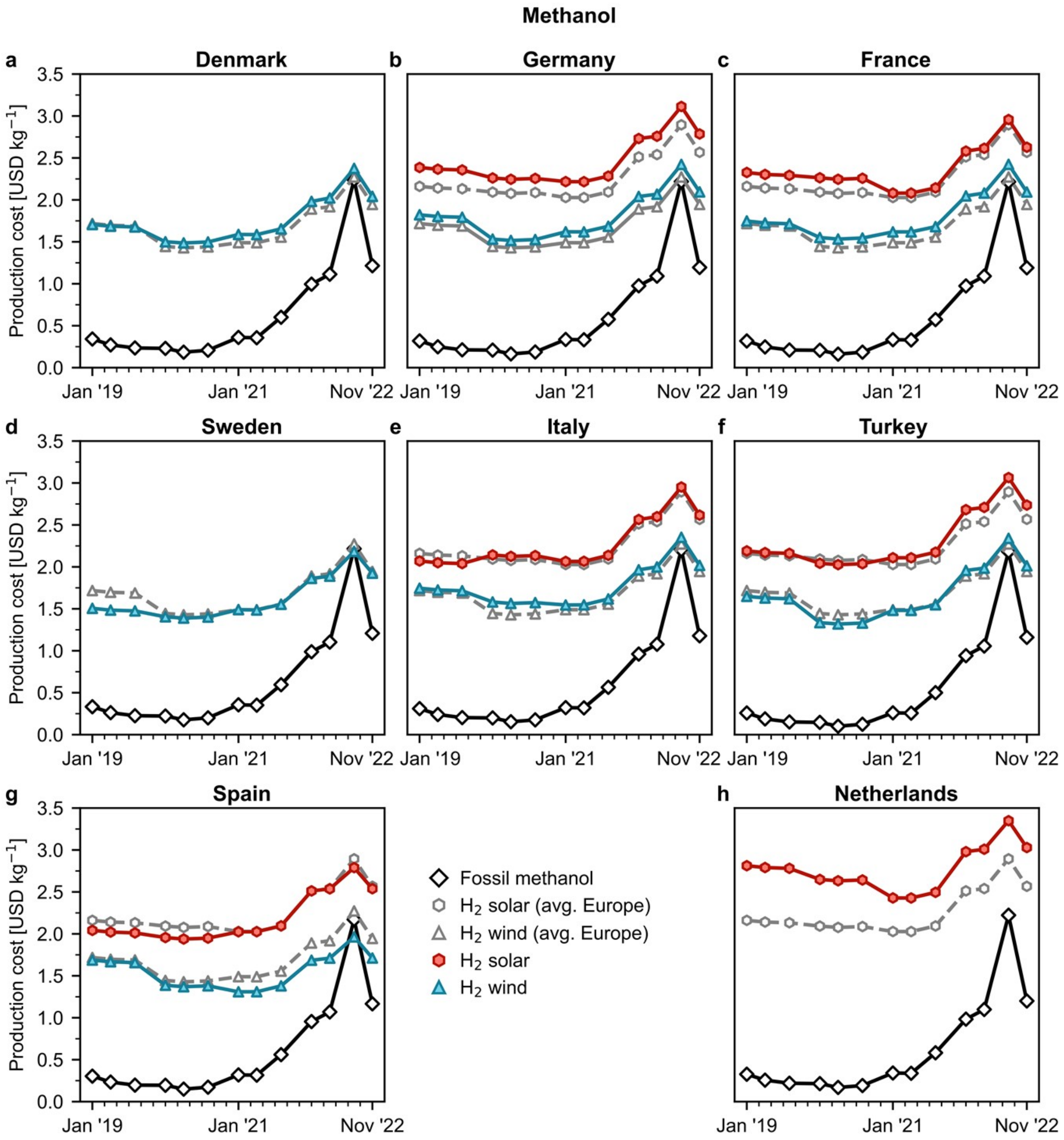

Figure S10. European regional production costs for fossil and green methanol in (a) Denmark, (b) Germany, (c) France, (d) Sweden, (e) Italy, (f) Turkey, (g) Spain, and (h) Netherlands. The levelised cost of electricity and hydrogen used to calculate the production cost are shown in **Table S9** for solar and **Table S10** for wind, respectively. Access to solar data for Denmark and Sweden and wind data for the Netherlands was unavailable. The fossil cost estimates are based on the Dutch TTF index for natural gas spot prices, as shown in **Figure 1** of the main manuscript.

5.2. Europe *vs* United States

Similar to Europe, we conducted cost estimations for both fossil and green ammonia and methanol in the United States (US). These values were then compared to the corresponding estimates for Europe, and the results are illustrated in **Figure S11.** For the fossil routes, the monthly average natural gas spot prices were extracted from the World Bank[7], while the grid electricity prices were obtained from the US Energy Information Administration.[13] Regarding the green routes, the Levelised Cost of Electricity (LCOE) data for 2019-2021 were extracted from IRENA. These values are presented in **Table S9** for solar and **Table S10** for wind, respectively. To calculate the Levelised Cost of Hydrogen (LCOH) for the US, a similar procedure as described in Section 2.4 was employed.

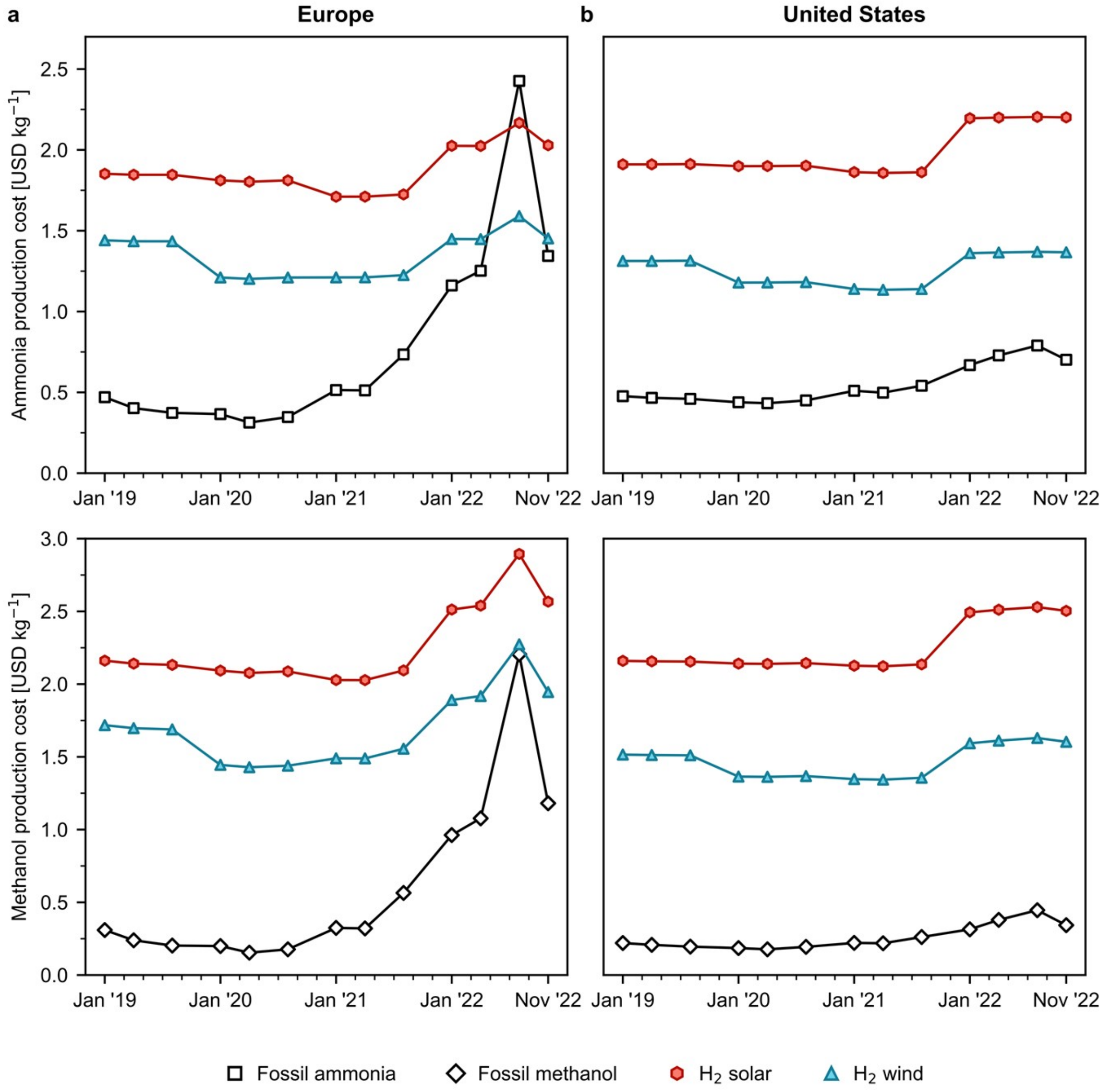

Figure S11. Production cost comparisons for ammonia and methanol in (a) Europe and (b) United States. In contrast to Europe, the green production pathways in the United States are economically non-competitive. This is primarily due to the fact that energy prices in the United States were not significantly affected by the energy crisis.

6. Additional results

Figure S12 shows the visual comparison of all the ammonia and methanol production routes, including hydrogen from grid electricity-powered water electrolysis. The levelised cost of grid-powered hydrogen is calculated using the levelised cost of electricity shown in **Figure S5**.

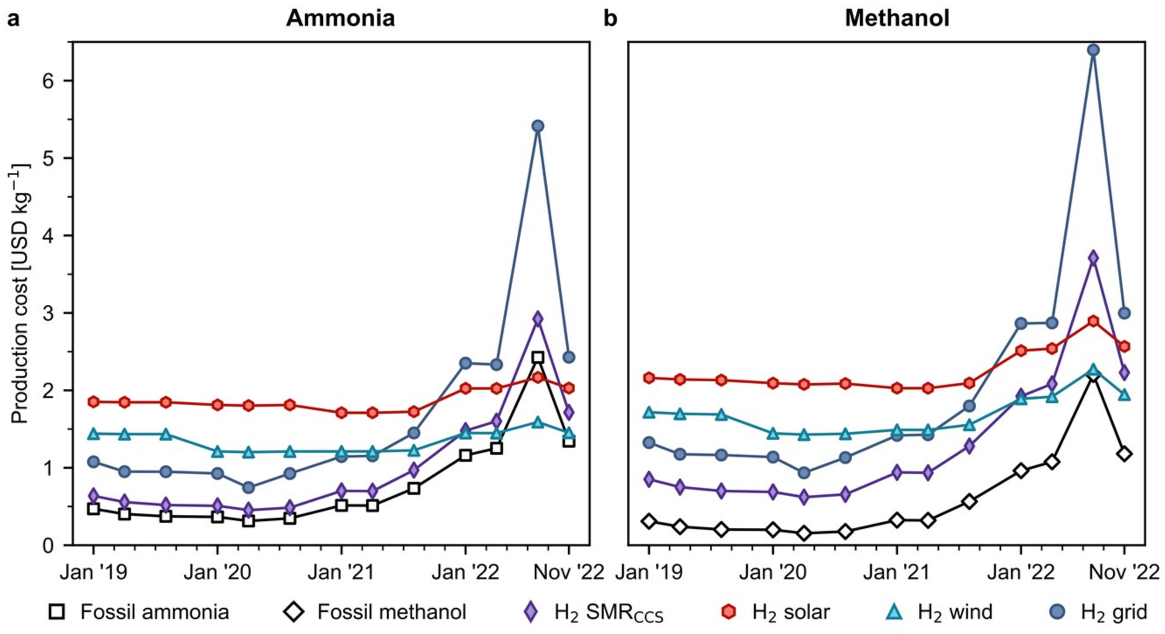

Figure S12. Production cost comparison between different routes for (a) ammonia and (b) methanol production. The levelised cost of hydrogen used to calculate the production cost for the solar and on-shore wind routes are shown in **Table S7**. For ammonia and methanol production from electrolysis using grid electricity, the levelised cost of hydrogen is calculated using the price of the electricity mix, as shown in **Figure 1** and **Figure S5.**

For the sake of clarity in graphical depictions, some additional results were not included in the main manuscript. The production cost for both fossil and green routes of ammonia and methanol production throughout the entire period of discussion is presented in **Figure S13**.

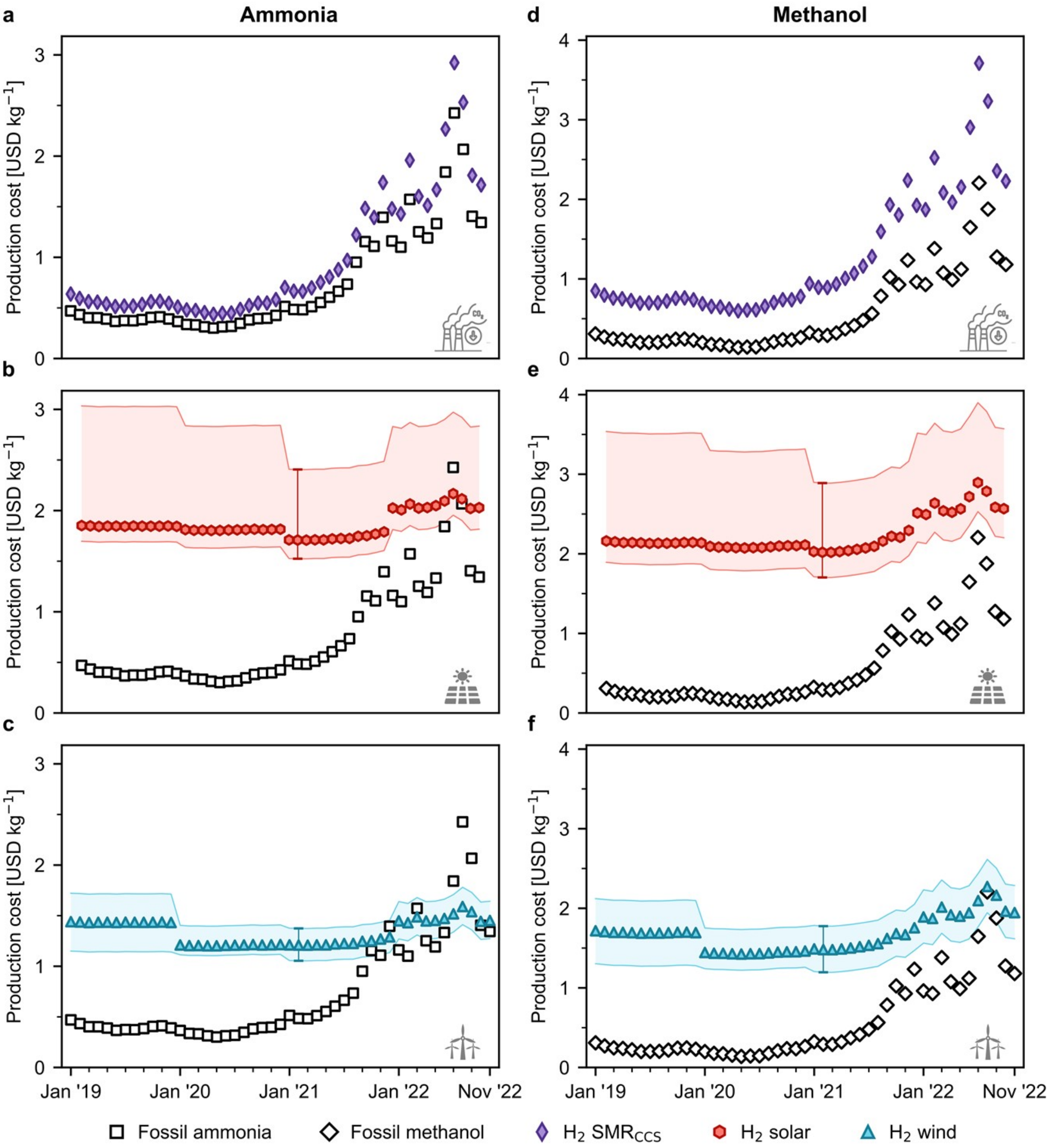

Figure S13. Production cost for green ammonia and methanol in Europe compared with the fossil counterpart. Green ammonia produced *via* the Haber-Bosch process with **(a)** blue hydrogen from steam methane reforming (SMR) coupled with carbon capture and storage (CCS) and green hydrogen from water electrolysis using **(b)** solar photovoltaic (PV) electricity or **(c)** on-shore wind electricity. Methanol synthesised from captured CO_2 and **(d)** blue hydrogen –SMR coupled with CCS–, **(e)** PV-based electrolytic hydrogen or **(f)** wind-based electrolytic hydrogen. The error bands indicate the lower and upper bounds for the levelised cost of hydrogen (see **Table S7**).

Figure S14 illustrates the cost breakdown for the production of ammonia and methanol through fossil and green routes. Of all the components, hydrogen makes the greatest contribution to the average production cost of green chemicals.

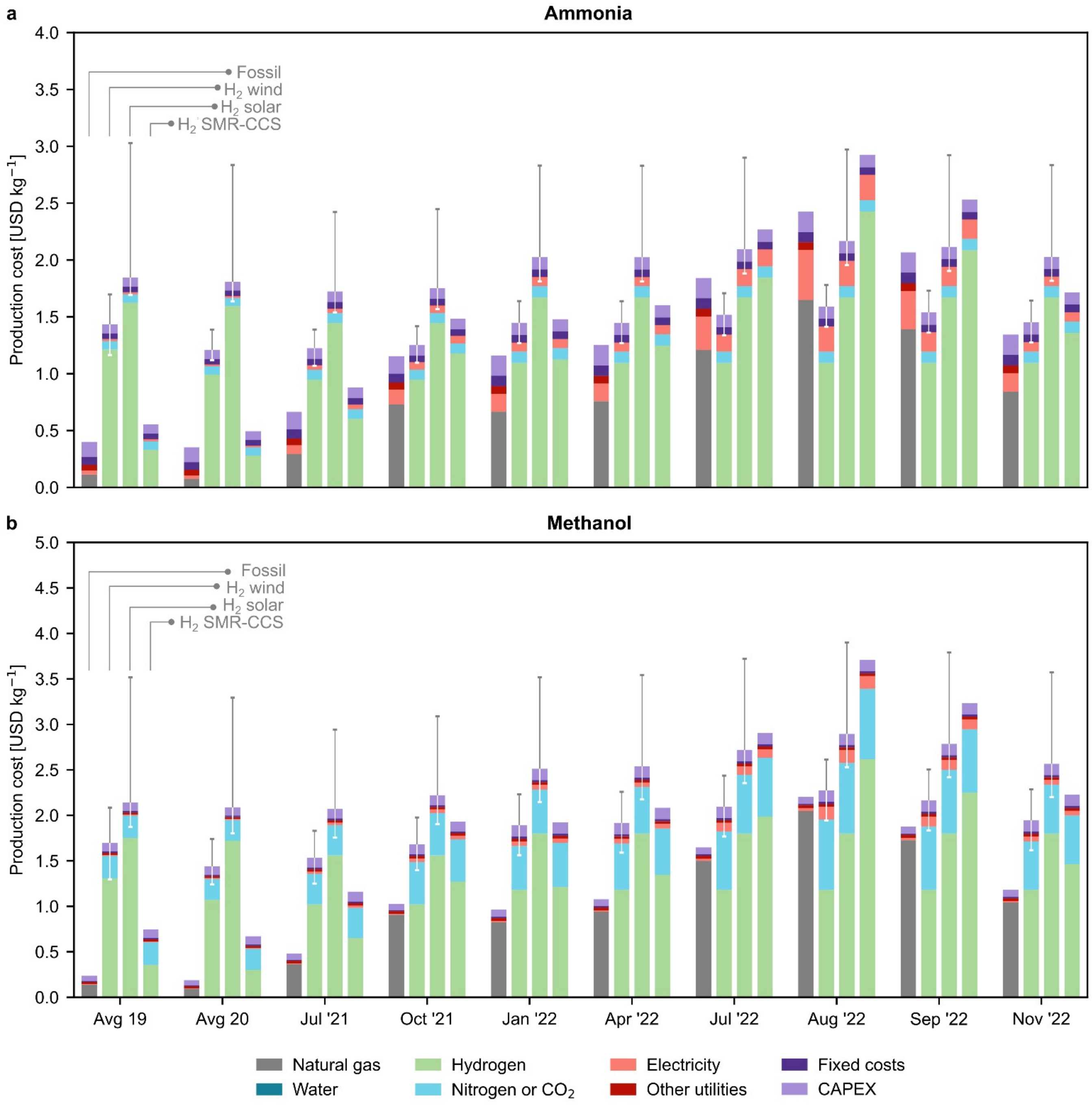

Figure S14. Breakdown of production costs for fossil and green ammonia (a) and methanol (b). Error bars display the production cost considering the lower and upper bound for the levelised cost of hydrogen.

Figure S15 shows the monthly average CO_2 from DAC prices. It can be seen that carbon dioxide capture prices are highly volatile and dependent on the price of natural gas and electricity. Around 0.1 kg of natural gas and 77 kWh of electric power is required per kg of CO_2 captured.

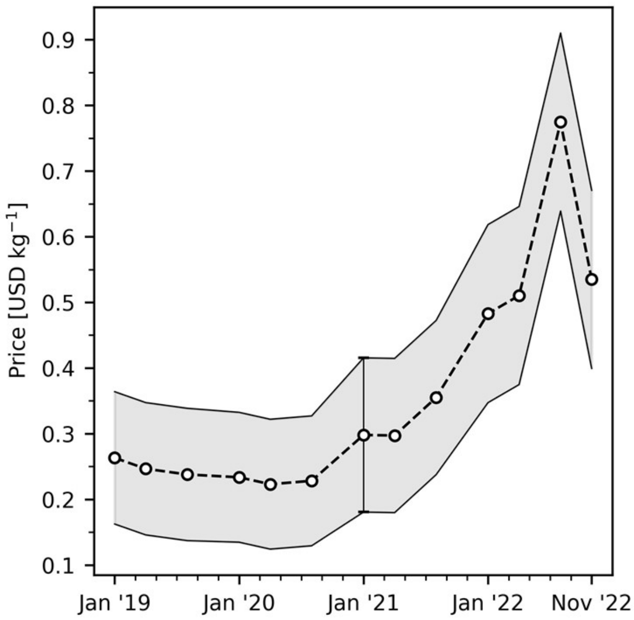

Figure S15. Evolution of carbon dioxide prices using DAC technology. Error bands display the capture cost considering the different Capital Recovery Factors (CRF) from the work done by Keith *et al.*[5]

Figure S16 shows the sensitivity analysis using future wind-based hydrogen costs and varying natural gas prices. The breakeven price of natural gas at which the green routes become economically viable is calculated using this analysis.

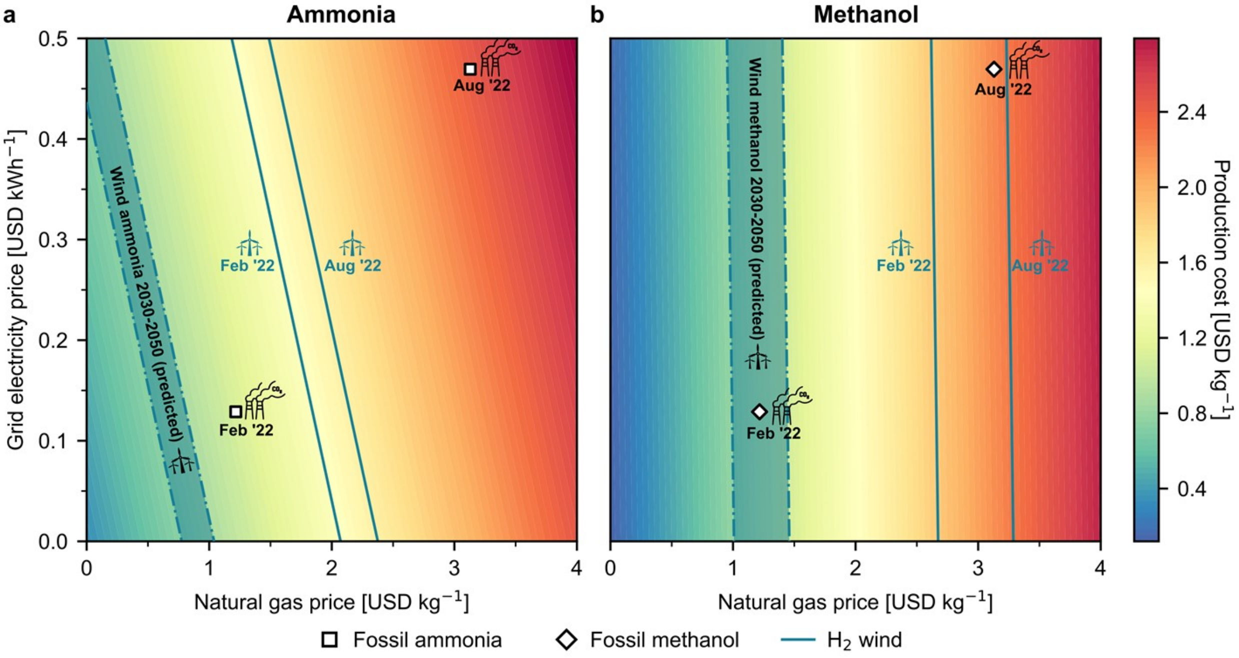

Figure S16. Sensitivity analysis of production cost estimates and future projections for (a) ammonia and (b) methanol production. The sensitivity analysis is conducted by varying the prices of natural gas and grid electricity to calculate the production cost estimates of the fossil routes. The scatter points, shown in black and white, depict the production cost range of fossil routes for 2022, with the minimum occurring in February and the maximum in August. The blue isolines in the contour plot illustrate the current production costs for the wind-based routes. Additionally, the blue-shaded region represents the projected costs for wind-based ammonia and methanol from 2030 to 2050.

Figure S17 shows the avoidance cost for the different routes of ammonia and methanol production including the lower and upper bounds of the hydrogen costs from **Table S7**.

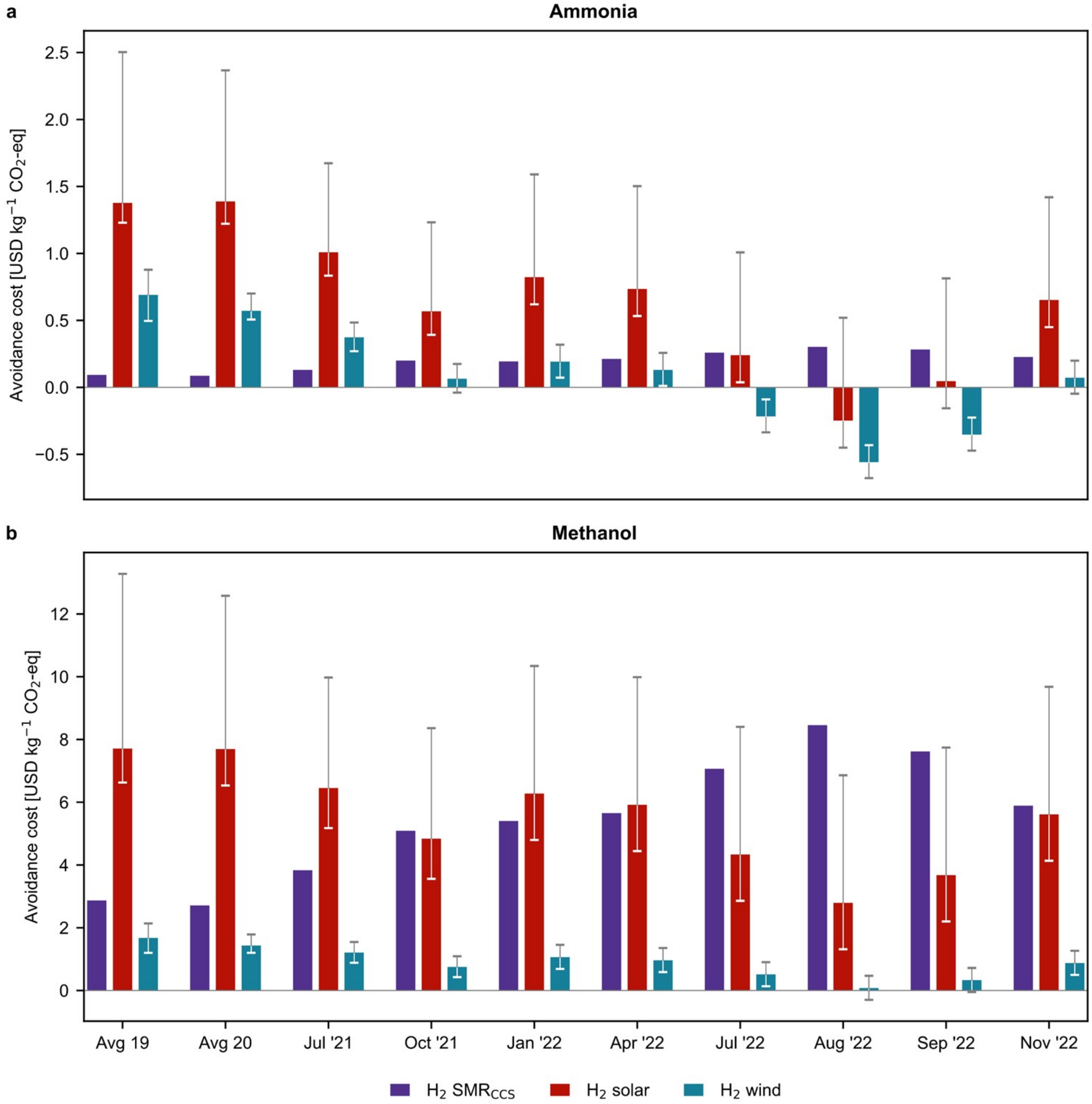

Figure S17. Avoidance costs for green (a) ammonia and (b) methanol production routes. Error bars display the production cost considering the lower and upper bound for the levelised cost of hydrogen (**Table S7**).

Due to insufficient data in the open literature, the levelised cost of hydrogen for the year 2022 is calculated by using the levelised cost of hydrogen from 2021 and adjusting it for inflation using the CEPCI, as reported in **Table S7**. Additionally, the increasing natural gas prices will result in a higher levelised cost of on-shore wind and solar electricity. Therefore, it is essential to examine how these natural gas prices impact the levelised cost of hydrogen during the period of elevated natural gas prices in 2022. To determine the percentage share of natural gas prices in relation to the levelised cost of electricity, we extract the quantity of natural gas required to generate 1 kWh of on-shore wind and solar electricity from their respective Ecoinvent inventories (refer to **Table S8** for the corresponding activity names of wind and solar electricity). Subsequently, we compare the levelised cost of hydrogen for 2022 under two scenarios: one where only inflation adjustments are made and another where both inflation adjustments and updated natural gas prices are considered. The findings are presented in **Figure S18**.

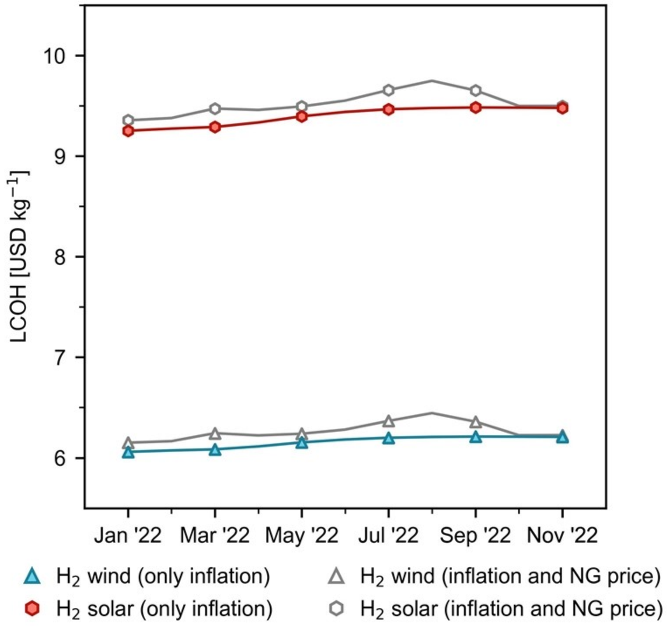

Figure S18. Levelised cost of hydrogen for 2022. The levelised cost of hydrogen for 2022 was calculated based on the levelised cost of hydrogen from 2021, considering two scenarios: one that includes only inflation adjustments (indicated by the coloured lines) and another that incorporates both inflation adjustments and updated natural gas prices (represented by the grey lines).

References

1 S. C. D'Angelo, S. Cobo, V. Tulus, A. Nabera, A. J. Martín, J. Pérez-Ramírez and G. Guillén-Gosálbez, *ACS Sustain. Chem. Eng.*, 2021, **9**, 9740–9749.

2 M. Appl, in *Ullmann's Encyclopedia of Industrial Chemistry*, ed. Wiley-VCH Verlag GmbH & Co. KGaA, Wiley-VCH Verlag GmbH & Co. KGaA, Weinheim, Germany, 2011, p. o02_o11.

3 A. González-Garay, M. S. Frei, A. Al-Qahtani, C. Mondelli, G. Guillén-Gosálbez and J. Pérez-Ramírez, *Energy Environ. Sci.*, 2019, **12**, 3425–3436.

4 K. M. V. Bussche and G. F. Froment, *Methanol kinetics*, 1996, vol. 161.

5 D. W. Keith, G. Holmes, D. St. Angelo and K. Heidel, *Joule*, 2018, **2**, 1573–1594.

6 G. P. Towler and R. K. Sinnott, *Sinnott & Towler*, Butterworth-Heinemann, 2013.

7 The World Bank, *Commodity Markets Outlook: The Impact of the War in Ukraine on Commodity Markets*, The World Bank Group, Washington DC, 2022.

8 P. Heather, *European Traded Gas Hubs: the supremacy of TTF*, The Oxford Institute for Energy Studies, 2022.

9 EMBER, European power price tracker, https://ember-climate.org/data/data-tools/europe-power-prices/, (accessed February 23, 2023).

10 B. Parkinson, P. Balcombe, J. F. Speirs, A. D. Hawkes and K. Hellgardt, *Energy Environ. Sci.*, 2019, **12**, 19–40.

11 IRENA, *Renewable power generation costs in 2021*, International Renewable Energy Agency, 2021.

12 G. Wernet, C. Bauer, B. Steubing, J. Reinhard, E. Moreno-Ruiz and B. Weidema, *Int. J. Life Cycle Assess.*, 2016, **21**, 1218–1230.

13 U.S. Energy Information Administration, Average retail price of electricity, United States, monthly, https://www.eia.gov/electricity/data/browser/#/topic/7?agg=2, (accessed May 24, 2023).
